# Unlocking liver physiology: comprehensive pathway maps for mechanistic understanding

**DOI:** 10.3389/ftox.2025.1619651

**Published:** 2025-07-07

**Authors:** Luiz Ladeira, Anouk Verhoeven, Jonas van Ertvelde, Jian Jiang, Alessio Gamba, Julen Sanz-Serrano, Tamara Vanhaecke, Harm J. Heusinkveld, Ramiro Jover, Mathieu Vinken, Liesbet Geris, Bernard Staumont

**Affiliations:** ^1^ Biomechanics Research Unit, GIGA Institute, University of Liège, Liège, Belgium; ^2^ Department of Pharmaceutical and Pharmacological Sciences, Entity of In Vitro Toxicology and Dermato-Cosmetology, Vrije Universiteit Brussel, Ixelles, Belgium; ^3^ Centre for Health Protection, National Institute for Public Health and the Environment (RIVM), Bilthoven, Netherlands; ^4^ Department of Biochemistry & Molecular Biology, Exp. Hepatology Joint Unit, University of Valencia, IIS Hospital La Fe, CIBERehd, Valencia, Spain; ^5^ Skeletal Biology and Engineering Research Center, KU Leuven, Leuven, Belgium; ^6^ Biomechanics Section, Department of Mechanical Engineering, KU Leuven, Leuven, Belgium

**Keywords:** physiological maps, toxicology, systems biology, hepatology, new approach methodologies

## Abstract

**Aims:**

*In silico* methods provide a resourceful toolbox for new approach methodologies (NAMs). They can revolutionize chemical safety assessment by offering more efficient and human-relevant alternatives to traditional animal testing. In this study, we introduce two Liver Physiological Maps (PMs); comprehensive and machine-readable graphical representations of the intricate mechanisms governing two major liver functions.

**Methods:**

Two PMs were developed through manual literature curation, integrating data from established pathway resources and domain expert knowledge. Cell-type specificity was validated using Human Protein Atlas datasets. An interactive version is available online for exploration. Cross-comparison analysis with existing Adverse Outcome Pathway (AOP) networks was performed to benchmark physiological coverage and identify knowledge gaps.

**Results:**

The LiverLipidPM focuses on liver lipid metabolism, detailing pathways involved in fatty acid synthesis, triglycerides, cholesterol metabolism, and lipid catabolism in hepatocytes. And the LiverBilePM represents bile acid biosynthesis and secretion processes, detailing biosynthesis, transport, and secretion processes between hepatocytes and cholangiocytes. Both maps integrate metabolism with signaling pathways and regulatory networks. The interactive maps enable visualization of molecular pathways, linkage to external ontologies, and overlay of experimental data. Comparative analysis revealed unique mechanisms to each map and overlaps with existing AOP networks. Chemical-target queries identified new potential targets in both PMs, which might represent new molecular initiating events for AOP network extension.

**Conclusion:**

The developed liver PMs serve as valuable resources for hepatology research, with a special focus on hepatotoxicity, supporting the refinement of AOP networks and the development of human-oriented *in vitro* test batteries for chemical toxicity assessment. These maps provide a foundation for creating computational models and mode-of-action ontologies while potentially extending their utility to systems biology and drug discovery applications.

## 1 Introduction

The liver is a vital organ responsible for several essential functions in the human body, including metabolism, immunity, digestion, and detoxification of xenobiotics (Chiang, 2013; Boyer, 2013; Alves-Bezerra and Cohen, 2017; Verhoeven et al., 2024). Its unique dual blood supply from the portal vein and the hepatic artery allows it to interact with the endocrine and gastrointestinal systems, supporting several metabolic functions such as lipid metabolism. Additionally, the liver plays a crucial role in bile acid biosynthesis and secretion, which are vital for preserving the body’s homeostasis. Exposure to toxic substances can result in liver injury, including cholestasis, steatosis, fibrosis, and cancer (Gijbels and Vinken, 2017; Mellor, Steinmetz, and Cronin, 2016). Therefore, comprehensive understanding of the mechanisms that drive human liver functions is critical for advancing mechanistic-based risk assessment in toxicology. This knowledge can pave the way for developing more precise and human-centered approaches for identifying and evaluating chemical hazards and risks.

New approach methodologies (NAMs) for next generation risk assessment combine human-oriented *in vitro* and *in silico* methods, including artificial intelligence (AI) tools and mechanistic models, to unravel mechanisms of toxicity (Vinken et al., 2021). In this context, the Physiological Maps (PMs) framework provides the blueprint for molecular mechanistic understanding of toxicity processes, linking to specific disease mechanisms summarized into qualitative and quantitative adverse outcome pathway (AOP) networks, and serving as a biological foundation for the development of mode-of-action ontologies (Desprez et al., 2019). PMs are standardized and machine-readable graphical representations of molecular and cellular processes associated with specific cell and/or organ functions, including homeostatic processes (Staumont et al., 2025). Their development process is highly inspired by the Disease Maps (DMs) project (Mazein et al., 2018; Ostaszewski et al., 2019). While DMs mostly focus on representing disease mechanisms, PMs depict undisturbed physiology. They act as a knowledge repository that integrates relationships curated from a range of sources, including the literature and open access resources mapping pathways - such as Reactome (Milacic et al., 2024), KEGG (Kanehisa et al., 2023), Wikipathways (Agrawal et al., 2024) and DMs modules (Fujita et al., 2014; Ostaszewski et al., 2021; Serhan et al., 2020). Moreover, PMs are curated for cell- and/or organ-specific scenarios. Like DMs, PMs are dynamic tools where new knowledge is seamlessly integrated, resulting in the continuous generation of updated versions through a community-based effort. They are machine-readable, as they rely on a standardized Systems Biology Graphical Notation (SBGN) (Novère et al., 2009) and can therefore be stored in different systems biology file formats (e.g., SBML, GPML - explained in Box 1 - and others). Additionally, they are designed in a modular, interoperable, and reusable manner, making them adaptable for various cell-specific contexts, diseases, or physiological conditions and perturbations.

In the present article, we present the development of two PMs of human liver functions: the Liver Lipid Metabolism and the Liver Bile Secretion PMs (LiverLipidPM and LiverBilePM). We also include a reproducible method for AOP benchmarking against undisturbed physiological mechanisms and discuss their potential applications in toxicology and systems medicine.

## 2 Results and discussion

We developed two PMs, each covering an important liver function whose impairment can lead to the distinct clinical conditions of steatosis and cholestasis. Both liver pathologies can be caused by exogenous substances through various mechanisms.

**FIGURE 1:**
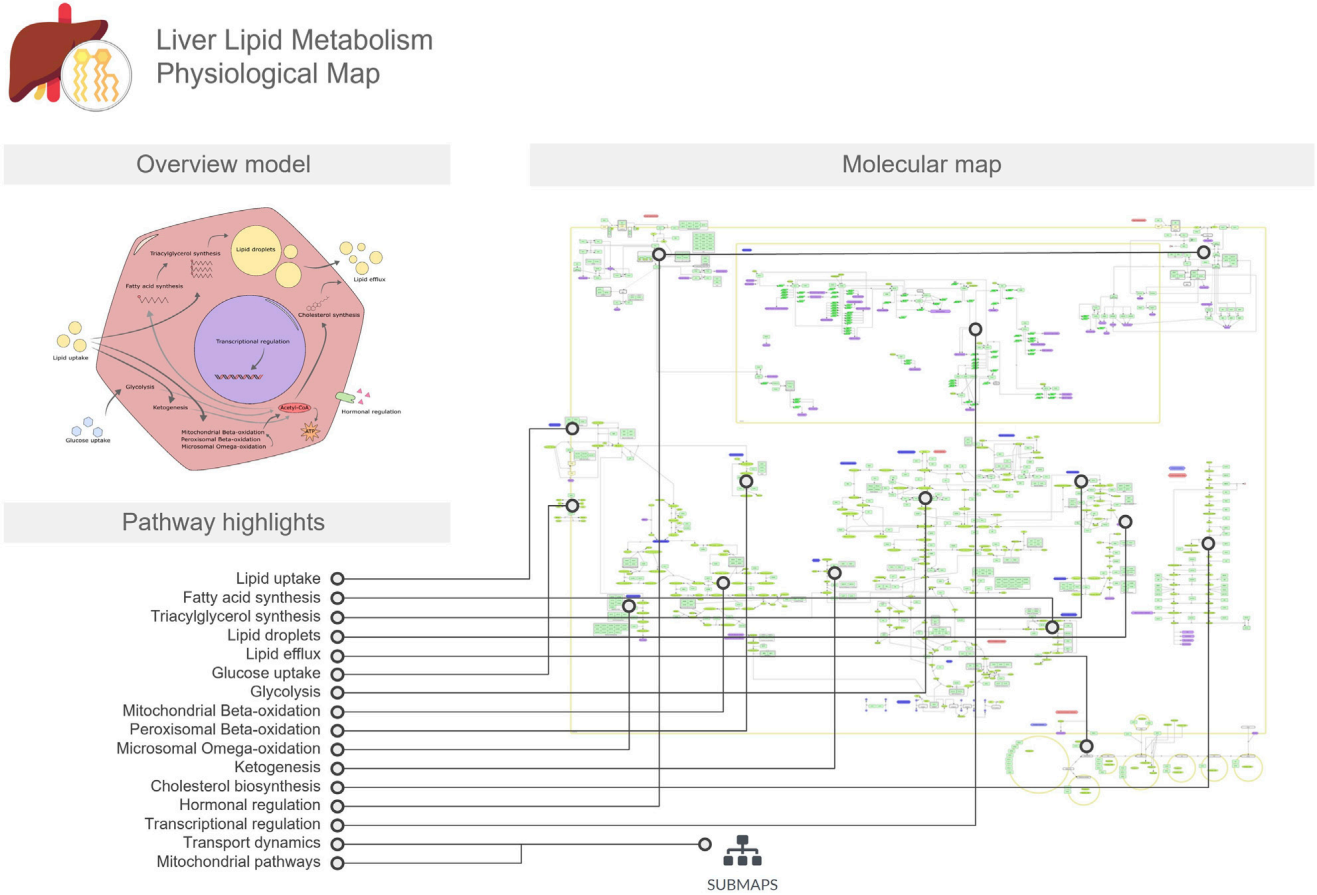
The Liver Lipid Metabolism Physiological Map (LiverLipidPM) is focused on lipid metabolism, including transport across hepatocyte membranes, lipid and cholesterol biosynthesis, and fatty acid oxidation. The overview model includes a conceptual illustration of these processes in the hepatocyte, serving as a mini-map to the molecular map. In the figure, we linked the pathways and mechanisms listed in the pathway highlights to the respective regions in which they are represented in the detailed molecular map.

The LiverLipidPM provides a detailed overview of the pathways involved in lipid metabolism (Figure 1). More specifically, the biological processes involved in the synthesis of fatty acids, triglycerides, and cholesterol, as well as their uptake and export mechanisms that facilitate access to the enzymes required for biotransformation processes. In addition, the map includes pathways related to lipid catabolism through mitochondrial and peroxisomal activities. A dedicated submap illustrates specific mitochondrial functions, such as reactive oxygen species scavenging and oxidative phosphorylation. The map also covers regulatory mechanisms that maintain lipid homeostasis through hormone signaling, transcription factor dynamics, and feedback loops. This PM depicts the complex network of biochemical reactions and molecular interactions occurring within a generic hepatocyte, represented by a single cellular compartment. To increase cell type specificity, the resource includes carefully curated proteins, genes, and ribonucleic acid (RNA) molecules known to be expressed in liver cells, validated against the Human Protein Atlas single cell datasets (Karlsson et al., 2021). This curation process ensures that the visualization accurately reflects the unique molecular landscape of hepatocytes, providing a comprehensive and tissue-specific representation of cellular processes in the liver.

**FIGURE 2:**
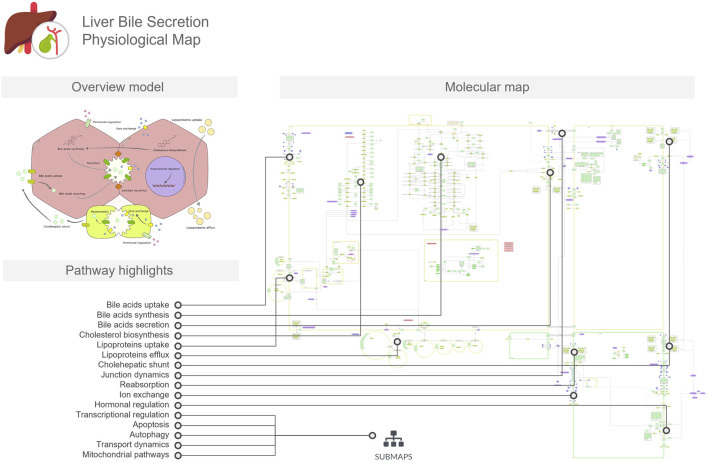
The Liver Bile Acids Secretion Physiological Map (LiverBileAcidsPM) focuses on bile acid biosynthesis in hepatocytes, transport across hepatocyte and cholangiocyte membranes, cholehepatic shunt, cell junction dynamics, and recycling processes. The overview model includes a conceptual illustration of these processes in the hepatocyte, serving as a mini-map to the molecular map. In the figure, we linked the pathways and mechanisms listed in the pathway highlights to the respective regions in which they are represented in the detailed molecular map.

The LiverBilePM provides a detailed overview of the biological pathways involved in the biosynthesis, transport, and secretion of bile acids in the liver and considers the interactive interface between hepatocytes and cholangiocytes through the bile canaliculi (Figure 2). This map also depicts cholesterol biosynthesis and metabolism, leading to bile acid biosynthesis and their subsequent transport across cellular membranes into the canaliculi spaces. It also includes pathways for lipoprotein uptake and efflux, as well as bile acid influx and recycling mechanisms, including the cholehepatic shunt. Besides that, regulatory control mechanisms through hormonal signaling, gene regulatory networks and adaptive tuning are also included. Hepatocytes and cholangiocytes are represented as four main compartments, two for each cell type, and a delimited space between two hepatocytes and two cholangiocytes represents a bile duct and bile canaliculus, respectively. As with the LiverLipidPM, cellular specificity was also taken into consideration, and map entities were curated using the Human Protein Atlas resources for both cell types presented on the LiverBilePM.

**FIGURE 3:**
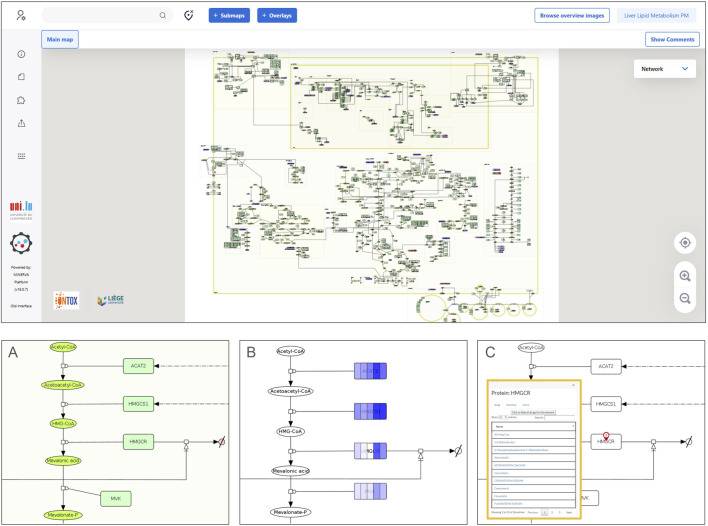
MINERVA platform visualization of a physiological map. The upper panel displays a full map visualization in the MINERVA platform interface. A zoom on a pathway (cholesterol biosynthesis) is depicted in **(A)**, showing the graphical representation of the molecular interactions using the Systems Biology Graphical Notation. Panel **(B)** shows how data can be visualized: a color gradient is used to represent a range of numerical values associated with each map entity. In this particular example, the intensity of the blue color indicates the level of RNA amounts in hepatocytes (darker shades represent higher levels) from different single-cell data clusters using a dataset from the Human Protein Atlas (proteinatlas.org) (Karlsson et al., 2021). Panel **(C)** shows the output of a drug query for a specific protein target (HMGCR), which retrieves results from DrugBank (Knox et al., 2024) and ChEMBL (Zdrazil et al., 2024).

Both maps integrate metabolism with signaling pathways and regulatory networks using a systems biology approach, as depicted in Figure 3A, and they were constructed utilizing manually curated human-relevant data. They both share 550 unique nodes identified by their HGNC approved symbols, which are mainly enriched for Reactome terms related to mitochondrial processes, such as aerobic respiration and respiratory electron transport, complex I biogenesis, and mitochondrial protein degradation, as well as metabolism of steroids and phase I metabolism of compounds. Supplementary Figure S1 shows each map entity frequency, their overlapping entities and the top 5 enriched Reactome terms for each resource. The Supplementary Material contains tables for each enrichment analysis (unique map terms and their overlapping processes). The PMs are designed to guide the development of mechanistic-based *in vitro* test batteries, *in silico* methods including AI approaches, and mode-of-action ontologies, all aimed at supporting the mechanistic prediction of chemical toxicities in humans (Vinken et al., 2021; Heusinkveld et al., 2021).

Additionally, the liver PMs can be applied to visually overlay omics data onto the pathways (Figure 3B) using, for example, the MINERVA (Molecular Interaction NEtwoRk VisuAlization) platform (Satagopam et al., 2016; Hoksza et al., 2020). This visualization resource allows for the exploration of variability in cell physiology by comparing different conditions side-by-side. For instance, Figure 3B illustrates five distinct clusters of hepatocytes from a Human Protein Atlas single-cell dataset, overlayed on a section of the cholesterol biosynthesis pathway. By leveraging the MINERVA platform features, it is possible to query external ontologies for chemicals and drugs that interact with molecular targets present in the maps (Figure 3C). The interactive version of both liver PMs can be accessed and explored on the following MINERVA platform weblink: https://ontox.elixir-luxembourg.org/minerva/.

Furthermore, PMs serve as repositories of existing biological knowledge, which can be used to support the development of AOPs. Two recent efforts to map AOP networks for steatosis and cholestasis highlight how toxicity mechanisms interact at a higher mechanistic level (Van Ertvelde et al., 2023; Verhoeven et al., 2024). By providing a high-resolution molecular description of these mechanisms, PMs are valuable for benchmarking AOP network coverage of biological processes and identifying new molecular initiating event targets that lead to pathway perturbations and downstream toxicological key events up to organ phenotypic alterations. The cross-comparison analysis between each AOP network with their related PM (Steatosis AOP network vs. LiverLipidPM and Cholestasis AOP network vs. LiverBilePM) revealed a small overlapping rate (10.1% and 9.5% respectively for each comparison). Reactome enrichment for each specific gene list (unique for PMs, unique for AOP networks and their overlaps) revealed their related mechanisms (Supplementary Material), which are mostly related to mitochondrial processes, highlighting the central role of this organelle in both steatosis and cholestasis. In addition, a chemical-target search by querying DrugBank (Knox et al., 2024) and ChEMBL (Zdrazil et al., 2024) databases via the MINERVA platform for targets present uniquely in the physiological maps, but not currently present in the AOP networks, found new 248 targets in the LiverLipidPM (with 898 interacting compounds) and 159 targets in the LiverBilePM (with 284 interacting compounds). These target lists are a starting point for exploration in order to increase the AOP network coverage for other possible molecular initiating events that could lead to both adverse outcomes. Moreover, this type of analysis facilitates hypothesis generation for new AOPs, by highlighting possible new mechanisms, AOP network – and PMs – gaps, and contributing to their refinement, expansion, and validation of AOPs, AOP networks and the maps. Finally, they can also be used as a basis for developing *in silico* models that address specific questions, as demonstrated by the DMs community (Niarakis et al., 2024).

PMs are aligned with the FAIR principles of Findability, Accessibility, Interoperability, and Reusability (Wilkinson et al., 2016) and present a unique identifier upon storage in BioStudies (Sarkans et al., 2018) for each released version (https://www.ebi.ac.uk/biostudies/studies?query=ontox+physiological+map). They are open-source, publicly accessible, and completely reusable, either in their entirety or as adaptable modules. The MIRIAM (Minimal Information Requested In the Annotation of biochemical Models) annotations (Juty, Le Novere, and Laibe, 2012) enhance the link between PMs and external databases for each node in the network. By incorporating annotated literature and pathway resources into the edges of the map, it enhances confidence and traceability in the information being presented. This ultimately increases the overall transparency of the data. The accessibility and reusability of these PMs promote collaboration and knowledge sharing within the scientific community, fostering advancements in curation efforts to expand these resources.

### 2.1 Challenges and future directions

The Liver PMs, while extensive, do not capture all known molecular processes related to the liver functions. This limitation stems from the manual curation process, which, despite expert involvement, is inherently constrained by time and resources. To enhance these maps, we plan to explore AI-assisted systematic review methods (Verhoeven et al., 2024; Van Ertvelde et al., 2023; Bozada et al., 2021) in the literature selection phase, and text mining and natural language processing techniques in the curation phase (Corradi et al., 2022; Bachman, Gyori, and Sorger, 2023). While AI-driven data extraction from text still faces challenges, such as avoiding AI-generated hallucinations, this level of automation can complement manual validation efforts.

By utilizing large-scale data analysis and machine learning techniques, we can discover novel molecular relationships and expand the resource’s detail and coverage, with the goal of more accurately describing human physiology. Examining differentially expressed genes across variations in standard physiological conditions (e.g., gender, age, populations, genotypic variations) can help to illuminate the mechanistic differences leading to diverse outcomes upon therapy administration or chemical exposure. Additionally, data-driven approaches for reconstructing mechanistic pathways (Miagoux et al., 2021) can help to address gaps in our understanding of human physiology.

To support research into chemical-induced toxicity endpoints, both PMs were specifically developed as tools fit for this purpose. However, the fact that they are modular and interoperable makes them valuable assets for the broader hepatology community, extending their usefulness beyond the scope of toxicology.

## 3 Conclusion

The Liver PMs were primarily designed to serve as a valuable resource for toxicology research. They were built to guide the refinement of AOP networks, enhance our understanding of human physiological mechanisms, and support the establishment of human-oriented *in silico* and *in vitro* test batteries for chemical toxicity assessment. Additionally, these maps were also intended to provide a rationale for creating dynamic computational models and to lay the groundwork for mode-of-action ontologies and mechanistic AI tools in toxicology. Beyond their initial focus, the Liver PMs may also be applicable to systems biology and drug discovery. As research progresses, these maps could become valuable in various aspects of pharmaceutical development, including drug repurposing efforts.

## 4 Methods

**FIGURE 4:**
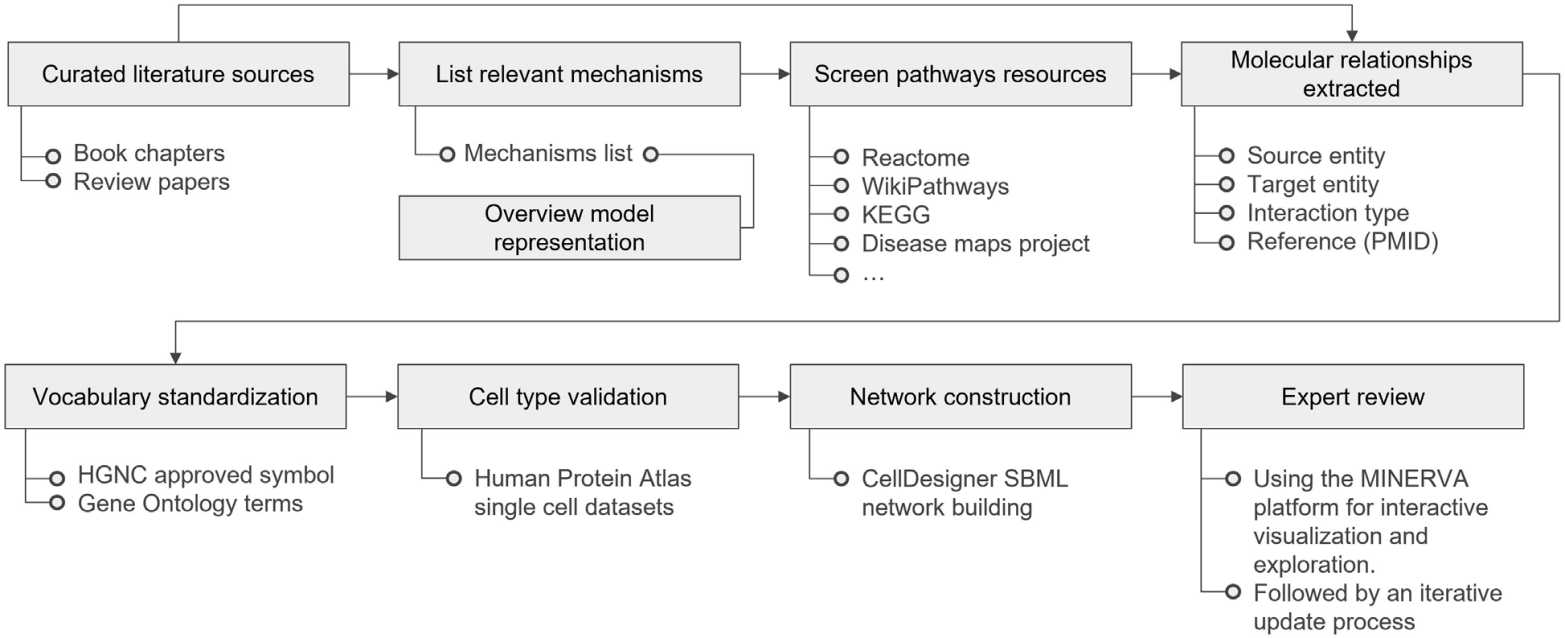
Physiological Maps curation workflow, from literature curation to expert review. KEGG stands for Kyoto Encyclopedia of Genes and Genomes; PMID for PubMed Identifier; HGNC for HUGO (Human Genome Organization) Gene Nomenclature Committee; SBML for Systems Biology Graphical Notation; and MINERVA for Molecular Interaction NEtwoRk VisuAlization.

The establishment of the PMs involves several steps: literature selection and curation, overview model representation, pathway resource screening, extraction of molecular relationships, nomenclature standardization, cell type curation, network diagramming, and expert review. Figure 4 highlights the entire workflow, detailing the key resources used in each phase. Methods for the PMs and AOP network comparison and overlay preparation, as well as a detailed description for the PMs cross-comparison analysis can be found in the Supplementary Material.

### 4.1 Data curation

To build the PMs, domain experts reviewed relevant literature, encompassing review papers and book chapters. The initial list of selected literature is included in the references of the maps planning documents (Supplementary Material). Mechanisms identified in the selected literature were compiled into a list, and key terms from this list were incorporated into an overview model (Supplementary Material). Pathways from established resources such as Reactome (Gillespie et al., 2022), KEGG (Kanehisa et al., 2023), Wikipathways (Martens et al., 2021) and DMs reusable modules (Fujita et al., 2014; Ostaszewski et al., 2021; Serhan et al., 2020) were screened for relevance, and pertinent models were extracted for further refinement and inclusion in the PMs. Different sources for each mechanisms represented are listed in each map table of contents (Supplementary Material). These sources served as the foundation for constructing the molecular diagrams. The process involved identifying molecular interactions within normal physiological processes, followed by extracting and detailing the causal relationships among them. Vocabulary was standardized using symbols approved by the HUGO (Human Genome Organization) Gene Nomenclature Committee (HGNC) (Seal et al., 2023) for genes, RNAs, and proteins, and Gene Ontology (The Gene Ontology Consortium et al., 2023) Biological Function terms for phenotypes where relevant. For cell type validation of specific isoforms of proteins, we used a single cell transcriptomic consensus dataset from the Human Protein Atlas - proteinatlas.org (Karlsson et al., 2021) (downloaded from https://www.proteinatlas.org/download/tsv/normal_ihc_cell_types.tsv.zip) to curate pathways relevant to a specific cell-type.

### 4.2 Graphical representation

The SBGN (Novère et al., 2009) Process Description (PD) language (Rougny et al., 2019) was the first choice of standard for pathway representation due to its ability to provide a high level of granularity, mechanistic insights, and a clear sequence of events. In instances where available information was sparse or insufficiently detailed for a full PD representation, a pragmatic approach was adopted. This consisted of combining SBGN Activity Flow (Mi et al., 2015) modules into the PD representation. This allowed for maintaining a balance between human readability and the need for flexibility when applying different analysis pipelines. The pathways and processes derived from literature or other pathway databases were represented manually in CellDesigner. When CellDesigner .XML files were available from other mapping projects, the pathways were directly reused and curated for relevance to the liver cells context.

### 4.3 Diagram editor and visualization platform

The maps were created and edited using the CellDesigner pathway editor (Funahashi et al., 2008). Graphical representation and literature annotation was done manually. For node annotation, we relied on HGNC approved symbol naming of the nodes and automated annotations using the MINERVA platform (Gawron et al., 2016; Hoksza et al., 2020) HGNC annotator, which annotates nodes with their respective Ensembl, Entrez gene, HGNC, RefSeq, UniProt identifiers. For the domain expert review phase, the MINERVA platform was employed. MINERVA’s well-structured commenting system, along with its map visualization and exploration capacities powered by the Google Maps API, facilitated the review process. To facilitate the use of MINERVA and to help the users to take full advantage of the platform, we have also prepared a video tutorial available at https://www.youtube.com/watch?v=CKKpAvSq560. A comprehensive user’s manual can be found in the developer’s webpage via https://minerva.pages.uni.lu/doc/manuals/.

### 4.4 Validation and standardization

The liver PMs’ validation strategy extends beyond traditional data curation approaches by integrating multiple validation layers. Each molecular component underwent cell-type specificity validation of gene expression and protein isoforms using the Human Protein Atlas resources (proteinatlas.org) (Karlsson et al., 2021), ensuring the biological relevance of the represented pathways for both hepatocytes and cholangiocytes. The SBGN (Novère et al., 2009) Process Description notation was selectively applied to pathways with strong mechanistic evidence, while Activity Flow representation was chosen for emerging mechanisms where evidence is still accumulating and in strategic decisions to enhance graphical representation by reducing diagram overload. This dual notation approach provides clear confidence indicators for map users, as Process Description sections reflect higher confidence in the mechanistic details. Notably, the iterative expert review process through MINERVA enabled the identification and resolution of potential inconsistencies between different pathway resources, particularly in cases where the sources (e.g., literature, Reactome, KEGG, WikiPathways and others) presented varying mechanistic details. When such discrepancies were encountered, additional literature validation was performed to determine the most accurate representation.

### 4.5 Documentation

To harness the full potential of PMs, a collaborative effort between domain experts and the curation team was undertaken to annotate and document the maps. This process involved the development of curation guidelines (Ladeira et al., 2024), coupled with comprehensive planning documents, as well as detailed tables of contents (Supplementary Material, and also available via https://github.com/ontox-maps/guides_and_documentation). We followed a comprehensive guide from the DMs community (Mazein et al., 2023), reinforcing adherence to the FAIR principles (Wilkinson et al., 2016). Metadata, including literature references, pathway resource references, and identifiers, can be found annotated directly into the SBML files of the maps. This approach not only facilitates the use of PMs within the toxicology ecosystem but also ensures that other researchers can seamlessly integrate and utilize these resources.

### 4.6 Physiological maps cross-comparison

Comparative analysis of the LiverLipidPM and LiverBilePM was performed to characterize their molecular composition and functional relationships. Molecular components were extracted from both maps using the minervar R package (version 0.8.15) (Gawron et al., 2023). Gene, protein, and RNA entities (all named with their relative HGNC approved symbol) were filtered and curated to generate gene lists for comparative analysis.

Set operations were applied to identify shared and unique molecular components between maps, with results visualized using Venn diagrams. Functional enrichment analysis was conducted using ReactomePA (Yu and He, 2016) to determine pathway enrichment for shared genes and genes unique to each map. Enrichment analysis results were combined and visualized as comparative bubble plots showing the top 5 enriched pathways for each gene set.

A detailed description of this section can be found in the supplementary text (Supplementary Material) as well as the gene lists and reproducible R scripts.

**Box 1:** Concepts and resource definitions.

Resource	Definition/comment
New Approach Methodologies (NAMs)	New approach methodologies (NAMs), are non-animal testing methods designed to reduce and replace existing traditional animal-based testing systems. Resource: https://www.oecd.org/chemicalsafety/testing/new-approach-methodologies-in-toxicology.htm
Disease Maps	Disease Maps are visual representations of disease mechanisms in a human- and machine-readable way. Each project in the Disease Maps community integrates molecular interactions and pathways involved in a particular pathological scenario. More recently, physiological maps and adverse outcome pathways have also been integrated into the Disease Maps project portfolio. Resource: https://disease-maps.org/
Systems Biology Graphical Notation (SBGN)	Systems Biology Graphical Notation: a standardized graphical representation of biological mechanisms. SBGN is composed of three different types of representations: Activity Flow, Process Description and Entity Relationships. Resource: https://sbgn.github.io/
SBGN Process Description	A type of SBGN representation in which a network is directed, sequential, and mechanistic. It allows an understanding of the temporal aspect of biochemical interactions. Resource: https://sbgn.github.io/specifications
SBGN Activity Flow	A type of SBGN representation in which a network is directed and sequential but not mechanistic at the molecular level. It shows the flow of information between biochemical entities, omitting information about how interactions occur, and is particularly convenient for representing the effects of perturbations. Resource: https://sbgn.github.io/specifications
HGNC approved symbol	HUGO (Human Genome Organization) Gene Nomenclature Committee: official gene names assigned by experts. Used for consistent gene identification. Resource: https://www.genenames.org/
Gene Ontology (GO) Biological Function	Gene Ontology Biological Function: standardized terms describing gene roles in organisms. Part of a larger system for classifying gene functions. Resource: http://geneontology.org/
Reactome	Reactome is a large database of expert-curated biological pathways and reactions. Provides visualization and analysis tools for these processes Resource: https://reactome.org/
WikiPathways	WikiPathways is a community-curated biological pathway database. Allows researchers to contribute and edit pathway information. Resource: https://www.wikipathways.org/
Kyoto Encyclopedia of Genes and Genomes (KEGG)	Kyoto Encyclopedia of Genes and Genomes is a database of genetic and molecular information, including pathway resources. Focuses on the systemic functions of genes and molecules. Resource: https://www.genome.jp/kegg/
Systems Biology Markup Language (SBML)	Systems Biology Markup Language: standard format for representing biological models in a machine-readable manner. Facilitates the exchange of models between different software tools. Resource: http://sbml.org/
Graphical Pathway Markup Language (GPML)	Graphical Pathway Markup Language: the WikiPathways standard format for representing biological models in a machine-readable manner. Facilitates the exchange of models between different software tools. Resource: https://pathvisio.org/documentation/GPML2021-doc.html

## Data Availability

The datasets presented in this study can be found in online repositories. The names of the repository/repositories and accession number(s) can be found below: The dataset is available in the BioStudies database (http://www.ebi.ac.uk/biostudies) under accession numbers S-ONTX35 (LiverBilePM) & S-ONTX36 (LiverLipidPM). All the maps are available at our GitHub organization (https://github.com/ontox-maps) and at the ONTOX MINERVA platform (https://ontox.elixir-luxembourg.org/minerva/), under the license Creative Commons Attribution 4.0 International (CC BY 4.0) License (https://creativecommons.org/licenses/by/4.0/).

## Abbreviations

AI, Artificial Intelligence; AOP, Adverse Outcome Pathway; DM, Disease Map; FAIR, Findable, Accessible, Interoperable, and Reusable; GO, Gene Ontology; GPML, Graphical Pathway Markup Language; HGNC, HUGO Gene Nomenclature Committee; HUGO, Human Genome Organization; KEGG, Kyoto Encyclopedia of Genes and Genomes; LiverBilePM, Liver Bile Secretion Physiological Map; LiverLipidPM, Liver Lipid Metabolism Physiological Map; MINERVA, Molecular Interaction NEtwoRk VisuAlization; MIRIAM, Minimal Information Requested In the Annotation of biochemical Models; NAM, New Approach Methodology; ONTOX, Ontology-driven and artificial intelligence-based repeated dose toxicity testing of chemicals for next generation risk assessment; PD, Process Description; PM, Physiological Map; PMID, PubMed Identifier; RNA, Ribonucleic Acid; SBGN, Systems Biology Graphical Notation; SBML, Systems Biology Markup Language.
