# Supplementary material for: Unlocking liver physiology: comprehensive pathway maps for mechanistic understanding
Source: Front Toxicol. 2025 Jul 7;7:1619651. doi: 10.3389/ftox.2025.1619651 (PMC12277266; doi:10.3389/ftox.2025.1619651)
Supplement: Supplementary file 1 [file Supplementaryfile1.zip › Supplementary Information/Liver_Bile_Secretion_PM_Planning_document.pdf]

https://ontox-project.eu/

Liver Bile Secretion Physiological Map

Planning document

Contents

1. Map setup **2**
    - 1.1. Development team 2
    - 1.2. Domain experts 2
    - 1.3. Map purpose 2
    - 1.4. Scope 3
    - 1.5. Granularity 3
    - 1.6. Sustainability plan 3
    - 1.7. Resources/funding 4
2. Map content **4**
    - 2.1. Cell types involved 4
    - 2.2. Pathways involved 4
    - 2.3. Molecules involved 4
    - 2.4. Modules/hallmarks 5
3. References **5**

University of Liège ONTOX's team

1. Map setup

This section outlines the main decisions for map setup, including the map's purpose, its scope, and the data model chosen.

1.1. Development team

Name	Affiliation	Contact	Role
Luiz Carlos Maia Ladeira	Université de Liège	lcladeira@uliege.be	Developer & curator
Alessio Gamba	Université de Liège	agamba@uliege.be	Curator
Bernard Staumont	Université de Liège	b.staumont@uliege.be	Curator
Raphaëlle Lesage	Université de Liège	raphaelle@vph-institute.org	Curator
Liesbet Geris	Université de Liège	liesbet.geris@uliege.be	PI

1.2. Domain experts

Name	Affiliation	Contact	Role
Mathieu Vinken	Vrije Universiteit Brussel	mathieu.vinken@vub.be	Domain expert
Tamara Vanhaecke	Vrije Universiteit Brussel	Tamara.Vanhaecke@vub.be	Domain expert
Julen Sanz Serrano	Vrije Universiteit Brussel	julen.sanz.serrano@vub.be	Domain expert
Annika Drees	Vrije Universiteit Brussel	annika.hanna.drees@vub.be	Domain expert
Anouk Verhoeven	Vrije Universiteit Brussel	Anouk.Verhoeven@vub.be	Domain expert
Jian Jiang	Vrije Universiteit Brussel	jian.jiang@vub.be	Domain expert
Jonas van Ertvelde	Vrije Universiteit Brussel	Jonas.Werner.E.Van.Ertvelde@vub.be	Domain expert
Ramiro Jover	Universitat De Valencia	Ramiro.jover@uv.es	Domain expert
Anna Rapisarda	Universitat De Valencia	anna.s.rapisarda@uv.es	Domain expert

1.3. Map purpose

Within the ONTOX project, we are designing a total of five Physiological Maps (PMs) describing physiological processes in the liver, the kidney, and the developing brain. These PMs are then used to assess relevant mechanistic coverage and the relationship between a specific organ function and a toxicological endpoint.

They are focused on describing the following physiological processes: bile secretion and lipid metabolism (liver); nephron physiology (kidney); neural tube closure (an update of the work of Heusinkveld et al., 2021, DOI: 10.1016/j.reprotox.2020.09.002); and brain development (brain).

These PMs will be used for exploring curated literature, analysing networks, and benchmarking the development of new Adverse Outcome Pathways (AOPs). These PMs are also the basis for developing quantitative disease ontologies, integrating different layers of pathological and toxicological information, chemical information (drug-induced pathways), and kinetic data. The resulting chemical-induced disease ontologies will provide a multi-layered platform for the integration and visualisation of such information. The ontologies will contribute to improving

understanding of organ/disease related pathways in response to chemicals, visualising omics datasets, developing quantitative methods for computational disease modelling and predicting toxicity, setting up an in vitro and in silico test battery to detect a specific type of toxicity, and developing new animal-free approaches for next-generation risk assessment.

1.4. Scope

The Liver Bile Secretion PM is intended to map the core bile acid synthesis, secretion, reabsorption and metabolism pathways and all the important secondary pathways that regulate and play key roles in this process. This map will be used to study chemical-induced cholestasis, and for this end, we aim to cover all important physiological pathways related to the disease development. The map encompasses all processes critical to triggering the corresponding Adverse Outcome Pathway (AOP) network on cholestasis (Gijbels et al., 2020, DOI: 10.1007/s00204-020-02691-9; Vinken et al., 2013, DOI: 10.1093/toxsci/kft177), including drug transporters and tight junction functionality, bile canaliculi dynamics, as well as bile homeostasis. However, it is not limited to the current available AOPs, extending to other physiological mechanisms important in cholesterol synthesis, bile production, secretion, reabsorption, and recycling. This map only depicts liver processes on the metabolism of bile acids.

This scope describes and limits the first version of the map. In future versions, additional pathways could be considered based on the data and literature analyzed.

1.5. Granularity

The Liver Bile Secretion PM is constructed using the Process Description SBGN language as the first choice for representing the pathways. Activity Flow SBGN can be found on the map only when there is a lack of information to describe essential connections in detail. In addition, Activity Flow can be used to improve human readability.

The map contains a top-level view represented by a graphical conceptual model. This model will be displayed on the MINERVA platform in the “Overview” box, and will contain an annotation file with coordinates to make the graphical conceptual model interactive, linking elements on this model to the SBGN map.

The main map will be constructed as an assembly of smaller submaps. Each submap will represent an individual pathway or a small group of pathways. This will allow for more efficient curation and handling of the pathways by the curation and domain expert teams.

1.6. Sustainability plan

All stable versions of the ONTOX maps are stored on BioStudies (https://www.ebi.ac.uk/biostudies/) with all the documentation and metadata provided.

The interim versions (under development versions) will be maintained on the ONTOX GitHub repository (https://github.com/ontox-maps/).

The maps can also be found on the MINERVA platform for curation, data visualisation, and exploration. The main link is https://ontox.elixir-luxembourg.org/minerva/, but each map has its own permanent link.

The University of Liège ONTOX team is responsible for developing, updating, and maintaining the maps.

1.7. Resources/funding

This project is funded by the European Union's Horizon 2020 research and innovation programme under grant agreement No 963845 (ONTOX project).

2. Map content

This part lists map components such as key molecules, pathways, and cell types involved, as well as outlines the larger-scale modules planned. Updated information about the current state of each pathway included on the map can be found in the map's Table of Contents.

2.1. Cell types involved

**Cell type**	**Identifier**
Hepatocyte	Cell Ontology: CL:0000182
Cholangiocyte	Cell Ontology: CL:1000488

2.2. Pathways involved

This map will contain these identified key mechanisms: bile salts and ions exchange, cholesterol biosynthesis, lipoprotein uptake, lipoprotein secretion, bile acid biosynthesis, canaliculi formation and contraction, canaliculi transport dynamics, bile salts secretion, bile salts circulation, bile salts uptake, cholehepatic shutting, hormonal signalling, and gene regulatory signalling.

2.3. Molecules involved

Key molecules represented on this map include bile acids, cholesterol, fatty acids, triglycerides, glycerol, the precursors and derivatives of the previously listed molecules, glucose, H_2O, glutathione,

bilirubin, ATP, secretin, somatostatin, glucose, nuclear receptors and other transcription factors, membrane transporters, enzymes, ions, and any molecule that could play a regulatory or catalytic role in any of the listed pathways. Clinical biomarkers can also be found. Exogenous chemicals, in general, are excluded from the scope of the PMs.

2.4. Modules/hallmarks

Bile salts and ions exchange, cholesterol biosynthesis, lipoprotein uptake, lipoprotein secretion, bile acids biosynthesis, canaliculi formation and contraction, canaliculi transport dynamics, bile salts secretion, bile salts circulation, bile salts uptake, cholehepatic shutting, hormonal signalling, gene regulatory signalling related to the following transcription factors: NR3C1, NR1B1, ESR1, RXRA, NR1I3, NR1H4, AHR, AHRR, PPARA, NFE2L2, FOXA2, NR1H3, PPARG, HNF4A, CREB1, PPARGC1A, MLXIPL, NR1I2, SREBF1, NR0B2, AKT2 and FOXO1.

3. References

The following references were provided by the domain experts as literature material for the PM's first version. They contain review papers and book chapters.

Arias, I. M. (Ed.). (2020). Section C: Transporters, Bile Acids, and Cholestasis. In *The liver: Biology and pathobiology* (Sixth edition). Wiley-Blackwell.

Boyer, J. L. (2013). Bile Formation and Secretion. In R. Terjung (Ed.), *Comprehensive Physiology* (1st ed., pp. 1035–1078). Wiley. https://doi.org/10.1002/cphy.c120027

Boyer, J. L., & Soroka, C. J. (2021). Bile formation and secretion: An update. *Journal of Hepatology*, *75*(1), 190–201. https://doi.org/10.1016/j.jhep.2021.02.011

Chiang, J. Y. L. (2013). Bile Acid Metabolism and Signaling. In R. Terjung (Ed.), *Comprehensive Physiology* (1st ed., pp. 1191–1212). Wiley. https://doi.org/10.1002/cphy.c120023

Dawson, P. A. (2016). Bile Acid Metabolism. In *Biochemistry of Lipids, Lipoproteins and Membranes* (pp. 359–389). Elsevier. https://doi.org/10.1016/B978-0-444-63438-2.00012-2

Dawson, P. A., & Karpen, S. J. (2015). Intestinal transport and metabolism of bile acids. *Journal of Lipid Research*, *56*(6), 1085–1099. https://doi.org/10.1194/jlr.R054114

Deferm, N., De Vocht, T., Qi, B., Van Brantegem, P., Gijbels, E., Vinken, M., de Witte, P., Bouillon, T., & Annaert, P. (2019). Current insights in the complexities underlying drug-induced cholestasis. *Critical Reviews in Toxicology*, *49*(6), 520–548. https://doi.org/10.1080/10408444.2019.1635081

Di Ciaula, A., Garruti, G., Lunardi Baccetto, R., Molina-Molina, E., Bonfrate, L., Wang, D. Q.-H., & Portincasa, P. (2017). Bile Acid Physiology. *Annals of Hepatology*, *16*, S4–S14. https://doi.org/10.5604/01.3001.0010.5493

Esteller, A. (2008). Physiology of bile secretion. *World Journal of Gastroenterology*, *14*(37), 5641. https://doi.org/10.3748/wjg.14.5641

Gijbels, E., Vilas-Boas, V., Deferm, N., Devisscher, L., Jaeschke, H., Annaert, P., & Vinken, M. (2019). Mechanisms and in vitro models of drug-induced cholestasis. *Archives of Toxicology*, *93*(5), 1169–1186. https://doi.org/10.1007/s00204-019-02437-2

Halilbasic, E., Claudel, T., & Trauner, M. (2013). Bile acid transporters and regulatory nuclear receptors in the liver and beyond. *Journal of Hepatology*, *58*(1), 155–168. https://doi.org/10.1016/j.jhep.2012.08.002

Li, T., & Chiang, J. Y. L. (2014). Bile Acid Signaling in Metabolic Disease and Drug Therapy. *Pharmacological Reviews*, *66*(4), 948–983. https://doi.org/10.1124/pr.113.008201

Minoru Kanehisa & Susumu Goto. (2020, June 4). *Bile secretion—Reference pathway* [Database]. KEGG. https://www.kegg.jp/entry/pathway+map04976

Minoru Kanehisa & Susumu Goto. (2021, January 14). *Primary bile acid biosynthesis—Reference pathway* [Database]. KEGG. https://www.kegg.jp/entry/pathway+map00120

Minoru Kanehisa & Susumu Goto. (2022, July 7). *Cholesterol metabolism* [Database]. KEGG. https://www.genome.jp/entry/map04979

Molinaro, A., Wahlström, A., & Marschall, H.-U. (2018). Role of Bile Acids in Metabolic Control. *Trends in Endocrinology & Metabolism*, *29*(1), 31–41. https://doi.org/10.1016/j.tem.2017.11.002

Russell, D. W. (2003). The Enzymes, Regulation, and Genetics of Bile Acid Synthesis. *Annual Review of Biochemistry*, *72*(1), 137–174. https://doi.org/10.1146/annurev.biochem.72.121801.161712

Šarenac, T. M., & Mikov, M. (2018). Bile Acid Synthesis: From Nature to the Chemical Modification and Synthesis and Their Applications as Drugs and Nutrients. *Frontiers in Pharmacology*, *9*, 939. https://doi.org/10.3389/fphar.2018.00939
