# Supplementary material for: Unlocking liver physiology: comprehensive pathway maps for mechanistic understanding
Source: Front Toxicol. 2025 Jul 7;7:1619651. doi: 10.3389/ftox.2025.1619651 (PMC12277266; doi:10.3389/ftox.2025.1619651)
Supplement: Supplementary file 1 [file Supplementaryfile1.zip › Supplementary Information/Liver_Bile_Secretion_PM_table_of_contents.pdf]

https://ontox-project.eu/

Liver Bile Secretion Physiological Map

Table of Contents

Pathway	Diagrams		Location on the map
	**Resource**	**Access**	
Cholesterol Biosynthesis Pathway	Reactome	10.3180/R-HSA-191273.7	Submap and main map
	WikiPathways	WP4718	
	Literature	Annotated on the map	
Fatty Acid and Cholesterol Transporters	WikiPathways	WP5061	Submap and main map
	WikiPathways	WP5304	
	KEGG	map04979	
	Literature	Annotated on the map	
Mitochondrial Metabolism Pathways	Parkinson's Disease Map	PDMap	Submap
	COVID19 Disease Map	COVID-19 Disease Map	
	WikiPathways	WP5175	
	WikiPathways	WP357	
	Literature	Annotated on the map	
Apoptosis Pathway	COVID19 Disease Map	COVID-19 Disease Map	Submap
	WikiPathways	WP254	
		WP1772	
	Literature	Annotated on the map	
Autophagy Pathway	Parkinson's Disease Map	PDMap	Submap
	WikiPathways	WP4923	
	Literature	Annotated on the map	
Glucagon signaling	Literature	Annotated on the map	Submap
Insulin signaling	Ageing Map	Ageing Map	Submap
	Literature	Annotated on the map	
Gene regulatory network	Literature	Annotated on the map	Submap and main map
	TRRUST dataset	TRRUST v2	
Bile salts circulation	Literature	Annotated on the map	Main Map
Bile salts uptake	Literature	Annotated on the map	Main Map
Cholehepatic shunt	Literature	Annotated on the map	Main Map
Canaliculi dynamics pathways	Literature	Annotated on the map	Main Map
Bile acids biosynthesis	KEGG	map00120	Main Map
	Literature	Annotated on the map	
Lipid droplet	WikiPathways	WP3901	Submap
