# Supplementary material for: Unlocking liver physiology: comprehensive pathway maps for mechanistic understanding
Source: Front Toxicol. 2025 Jul 7;7:1619651. doi: 10.3389/ftox.2025.1619651 (PMC12277266; doi:10.3389/ftox.2025.1619651)
Supplement: Supplementary file 1 [file Supplementaryfile1.zip › Supplementary Information/Liver_Lipid_Metabolism_PM_planning_document.pdf]

ONTOX https://ontox-project.eu/

Liver Lipid Metabolism Physiological Map

Planning document

Contents

1. Map setup **2**
  1.1. Development team 2
  1.2. Domain experts 2
  1.3. Map purpose 2
  1.4. Scope 3
  1.5. Granularity 3
  1.6. Sustainability plan 3
  1.7. Resources/funding 4
2. Map content **4**
  2.1. Cell types involved 4
  2.2. Pathways involved 4
  2.3. Molecules involved 4
  2.4. Modules/hallmarks 5
3. References **5**

Université de Liège team

1. Map setup

This section outlines the main decisions for map setup, including the map's purpose, its scope, and the data model chosen.

1.1. Development team

Name	Affiliation	Contact	Role
Luiz Ladeira	Université de Liège	lcladeira@uliege.be	Developer & curator
Alessio Gamba	Université de Liège	agamba@uliege.be	Curator
Bernard Staumont	Université de Liège	b.staumont@uliege.be	Curator
Raphaëlle Lesage	Université de Liège	raphaelle@vph-institute.org	Curator
Liesbet Geris	Université de Liège	liesbet.geris@uliege.be	PI

1.2. Domain experts

Name	Affiliation	Contact	Role
Mathieu Vinken	Vrije Universiteit Brussel	mathieu.vinken@vub.be	Domain expert
Tamara Vanhaecke	Vrije Universiteit Brussel	Tamara.Vanhaecke@vub.be	Domain expert
Jian Jiang	Vrije Universiteit Brussel	jian.jiang@vub.be	Domain expert
Anouk Verhoeven	Vrije Universiteit Brussel	Anouk.Verhoeven@vub.be	Domain expert
Julen Sanz Serrano	Vrije Universiteit Brussel	julen.sanz.serrano@vub.be	Domain expert
Annika Drees	Vrije Universiteit Brussel	annika.hanna.drees@vub.be	Domain expert
Jonas van Ertvelde	Vrije Universiteit Brussel	Jonas.Werner.E.Van.Ertvelde@vub.be	Domain expert
Ramiro Jover	Universitat De Valencia	Ramiro.jover@uv.es	Domain expert
Anna Rapisarda	Universitat De Valencia	anna.s.rapisarda@uv.es	Domain expert

1.3. Map purpose

Within the ONTOX project, we are designing a total of five Physiological Maps (PMs) describing physiological processes in the liver, the kidney, and the developing brain. These PMs are then used to assess relevant mechanistic coverage and the relationship between a specific organ function and a toxicological endpoint.

They are focused on describing the following physiological processes: bile secretion and lipid metabolism (liver); nephron physiology (kidney); neural tube closure (an update of the work of Heusinkveld et al., 2021, DOI: 10.1016/j.reprotox.2020.09.002); and brain development (brain).

These PMs will be used for exploring curated literature, analyzing networks, and benchmarking the development of new Adverse Outcome Pathways (AOPs). These PMs are also the basis for developing quantitative disease ontologies, integrating different layers of pathological and toxicological information, chemical information (drug-induced pathways), and kinetic data. The resulting chemical-induced disease ontologies will provide a multi-layered platform for integration and visualization of such information. The ontologies will contribute to improving understanding

of organ/disease related pathways in response to chemicals, visualizing omics datasets, developing quantitative methods for computational disease modeling and predicting toxicity, setting up an in vitro and in silico test battery to detect a specific type of toxicity, and developing new animal-free approaches for next-generation risk assessment.

1.4. Scope

The Liver Lipid Metabolism PM is intended to map the core lipid metabolism pathways and all the important secondary pathways that regulate and play key roles in this process. This map will be used to study chemical-induced steatosis, and for this end, we intend to explore key physiological mechanisms involved in disease development. We identified in collaboration with the domain experts the following mechanisms to be mapped: fatty acid uptake, fatty acid synthesis, triacylglycerol synthesis, cholesterol synthesis, and glycolysis (as inputs); mitochondrial beta-oxidation, peroxisomal beta-oxidation, microsomal omega-oxidation, ketogenesis, and very-low-density lipoprotein (VLDL) secretion (as outputs); hormones (insulin and glucagon); and transcriptional factors (as regulators).

This scope describes and limits the first version of the map. In future versions, additional pathways could be considered based on the data and literature analyzed.

1.5. Granularity

The Liver Lipid Metabolism PM is constructed using the Process Description SBGN language as the first choice for representing the pathways. Activity Flow SBGN can be found on the map only when there is a lack of information to describe essential connections in detail. In addition, Activity Flow can be used to improve human readability

The map contains a top-level view represented by a graphical conceptual model. This model will be displayed on the MINERVA platform in the “Overview” box, and will contain an annotation file with coordinates to make the graphical conceptual model interactive, linking elements on this model to the SBGN map.

The main map will be constructed as an assembly of smaller submaps. Each submap will represent an individual pathway or a small group of pathways. This will allow for more efficient curation and handling of the pathways by the curation and domain expert teams.

1.6. Sustainability plan

All stable versions of the ONTOX maps are stored on BioStudies (https://www.ebi.ac.uk/biostudies/) with all the documentation and metadata provided.

The interim versions (under development versions) will be maintained on the ONTOX GitHub repository (https://github.com/ontox-maps/).

The maps can also be found in the MINERVA platform for curation, data visualisation, and exploration. The main link is https://ontox.elixir-luxembourg.org/minerva/, but each map has its own permanent link.

The University of Liège ONTOX team is responsible for developing, updating, and maintaining the maps.

1.7. Resources/funding

This project is funded by the European Union's Horizon 2020 research and innovation programme under grant agreement No 963845 (ONTOX project).

2. Map content

This part lists map components such as key molecules, pathways, and cell types involved, as well as outlines the larger-scale modules planned. Updated information about the current state of each pathway included on the map can be found in the map's Table of Contents.

2.1. Cell types involved

Cell type	Identifier
Hepatocyte	Cell Ontology: CL:0000182

2.2. Pathways involved

This map will contain the identified key mechanisms: fatty acid uptake, fatty acid synthesis, triacylglycerol synthesis, cholesterol synthesis, and glycolysis (as input); mitochondrial beta-oxidation, peroxisomal beta-oxidation, microsomal omega-oxidation, ketogenesis, and very-low-density lipoprotein (VLDL) secretion (as output); hormones and transcriptional factors (as regulators).

2.3. Molecules involved

Key molecules represented on this map include fatty acids, triglycerides, glycerol, cholesterol, glucose, glycogen, other small carbohydrates, the precursors and derivatives of the previously listed molecules, insulin and glucagon, nuclear receptors and other transcription factors, membrane transporters, enzymes, ions, and any molecule that could play a regulatory or

catalytic role in any of the listed pathways. Clinical biomarkers can also be found. Exogenous chemicals, in general, are excluded from the scope of the PMs.

2.4. Modules/hallmarks

Fatty acid transporters, Fatty acid omega-oxidation, Fatty acid beta-oxidation, Ketogenesis, Peroxisomal beta-oxidation, Pentose phosphate metabolism, Glucolysis and Gluconeogenesis, Glycogen metabolism, TCA cycle, Electron Transport Chain, Cholesterol metabolism, Mitochondrial Fatty Acid Synthesis, Fatty acid elongation, Triacylglyceride Synthesis, Cholesterol biosynthesis pathway, Glucagon Signaling Pathway, Insulin Signaling Pathway, gene regulatory signaling related to the following transcription factors: NR3C1, NR1B1, ESR1, RXRA, NR1I3, NR1H4, AHR, AHRR, PPARA, NFE2L2, FOXA2, NR1H3, PPARG, HNF4A, CREB1, PPARGC1A, MLXIPL, NR1I2, SREBF1, NR0B2, AKT2 and FOXO1.

3. References

The following references were provided by the domain experts as literature material for the PM's first version. They contain review papers and book chapters.

Alaynick, W. A. (2008). Nuclear receptors, mitochondria and lipid metabolism. *Mitochondrion*, *8*(4), 329–337. https://doi.org/10.1016/j.mito.2008.02.001

Alves-Bezerra, M., & Cohen, D. E. (2017). Triglyceride Metabolism in the Liver. *Comprehensive Physiology*, *8*(1), 1–8. https://doi.org/10.1002/cphy.c170012

Bond, L. M., Miyazaki, M., O'Neill, L. M., Ding, F., & Ntambi, J. M. (2016). Fatty Acid Desaturation and Elongation in Mammals. In *Biochemistry of Lipids, Lipoproteins and Membranes* (pp. 185–208). Elsevier. https://doi.org/10.1016/B978-0-444-63438-2.00006-7

Brown, A. J., & Sharpe, L. J. (2016). Cholesterol Synthesis. In *Biochemistry of Lipids, Lipoproteins and Membranes* (pp. 327–358). Elsevier. https://doi.org/10.1016/B978-0-444-63438-2.00011-0

Foster, D. W. (2012). Malonyl-CoA: The regulator of fatty acid synthesis and oxidation. *The Journal of Clinical Investigation*, *122*(6), 1958–1959. https://doi.org/10.1172/jci63967

Gibbons, G. F., Wiggins, D., Brown, A.-M., & Hebbachi, A.-M. (2004). Synthesis and function of hepatic very-low-density lipoprotein. *Biochemical Society Transactions*, *32*(Pt 1), 59–64. https://doi.org/10.1042/bst0320059

Guillou, H., Martin, P. G. P., & Pineau, T. (2008). Transcriptional regulation of hepatic fatty acid metabolism. *Sub-Cellular Biochemistry*, *49*, 3–47. https://doi.org/10.1007/978-1-4020-8831-5_1

Guzmán, M., & Geelen, M. J. (1993). Regulation of fatty acid oxidation in mammalian liver. *Biochimica Et Biophysica Acta*, *1167*(3), 227–241. https://doi.org/10.1016/0005-2760(93)90224-w

Gyamfi, D., Ofori Awuah, E., & Owusu, S. (2019). Lipid Metabolism. In *The Molecular Nutrition of Fats* (pp. 17–32). Elsevier. https://doi.org/10.1016/B978-0-12-811297-7.00002-0

Horton, J. D., Goldstein, J. L., & Brown, M. S. (2002). SREBPs: Activators of the complete program of cholesterol and fatty acid synthesis in the liver. *The Journal of Clinical Investigation*, *109*(9), 1125–1131. https://doi.org/10.1172/JCI15593

Jump, D. B. (2011). Fatty acid regulation of hepatic lipid metabolism. *Current Opinion in Clinical Nutrition and Metabolic Care*, *14*(2), 115–120. https://doi.org/10.1097/MCO.0b013e328342991c

Kang, S., & Davis, R. A. (2000). Cholesterol and hepatic lipoprotein assembly and secretion. *Biochimica Et Biophysica Acta*, *1529*(1–3), 223–230. https://doi.org/10.1016/s1388-1981(00)00151-7

Kendrick, J. S., Wilkinson, J., Cartwright, I. J., Lawrence, S., & Higgins, J. A. (1998). Regulation of the assembly and secretion of very low density lipoproteins by the liver. *Biological Chemistry*, *379*(8–9), 1033–1040.

Lehner, R., & Quiroga, A. D. (2016). Fatty Acid Handling in Mammalian Cells. In *Biochemistry of Lipids, Lipoproteins and Membranes* (pp. 149–184). Elsevier. https://doi.org/10.1016/B978-0-444-63438-2.00005-5

Longo, N., Frigeni, M., & Pasquali, M. (2016). Carnitine transport and fatty acid oxidation. *Biochimica Et Biophysica Acta*, *1863*(10), 2422–2435. https://doi.org/10.1016/j.bbamcr.2016.01.023

McLeod, R. S., & Yao, Z. (2016). Assembly and Secretion of Triglyceride-Rich Lipoproteins. In *Biochemistry of Lipids, Lipoproteins and Membranes* (pp. 459–488). Elsevier. https://doi.org/10.1016/B978-0-444-63438-2.00016-X

Nelson, D. L., & Cox, M. M. (2017a). Fatty Acid Catabolism. In *Lehninger principles of biochemistry* (Seventh edition). W.H. Freeman and Company ; Macmillan Higher Education.

Nelson, D. L., & Cox, M. M. (2017b). Lipid Biosynthesis. In *Lehninger principles of biochemistry* (Seventh edition). W.H. Freeman and Company ; Macmillan Higher Education.

Nguyen, P., Leray, V., Diez, M., Serisier, S., Bloc'h, J. L., Siliart, B., & Dumon, H. (2008). Liver lipid metabolism. *Journal of Animal Physiology and Animal Nutrition*, *92*(3), 272–283. https://doi.org/10.1111/j.1439-0396.2007.00752.x

Park, C. Y., & Han, S. N. (2019). Lipid Pathway in Liver Cells and Its Modulation by Dietary Extracts. In *The Molecular Nutrition of Fats* (pp. 103–116). Elsevier. https://doi.org/10.1016/B978-0-12-811297-7.00008-1

Ridgway, N. D. (2016). Phospholipid Synthesis in Mammalian Cells. In *Biochemistry of Lipids, Lipoproteins and Membranes* (pp. 209–236). Elsevier. https://doi.org/10.1016/B978-0-444-63438-2.00007-9

Stahelin, R. V. (2016). Phospholipid Catabolism. In *Biochemistry of Lipids, Lipoproteins and Membranes* (pp. 237–257). Elsevier. https://doi.org/10.1016/B978-0-444-63438-2.00008-0

Tahri-Joutey, M., Andreoletti, P., Surapureddi, S., Nasser, B., Cherkaoui-Malki, M., & Latruffe, N. (2021). Mechanisms Mediating the Regulation of Peroxisomal Fatty Acid Beta-Oxidation by PPARα. *International Journal of Molecular Sciences*, *22*(16), 8969. https://doi.org/10.3390/ijms22168969

Titchenell, P. M., Lazar, M. A., & Birnbaum, M. J. (2017). Unraveling the Regulation of Hepatic Metabolism by Insulin. *Trends in Endocrinology & Metabolism*, *28*(7), 497–505. https://doi.org/10.1016/j.tem.2017.03.003

Wada, T., Gao, J., & Xie, W. (2009). PXR and CAR in energy metabolism. *Trends in Endocrinology & Metabolism*, *20*(6), 273–279. https://doi.org/10.1016/j.tem.2009.03.003

Ye, J., & DeBose-Boyd, R. A. (2011). Regulation of cholesterol and fatty acid synthesis. *Cold Spring Harbor Perspectives in Biology*, *3*(7), a004754. https://doi.org/10.1101/cshperspect.a004754
