# Supplementary material for: Unlocking liver physiology: comprehensive pathway maps for mechanistic understanding
Source: Front Toxicol. 2025 Jul 7;7:1619651. doi: 10.3389/ftox.2025.1619651 (PMC12277266; doi:10.3389/ftox.2025.1619651)
Supplement: Supplementary file 1 [file Supplementaryfile1.zip › Supplementary Information/Liver_Lipid_Metabolism_PM_table_of_contents.pdf]

ONTOX https://ontox-project.eu/

Liver Lipid Metabolism Physiological Map

Table of Contents

Pathway	Diagrams		Location on the map
	**Resource**	**Access**	
Cholesterol Biosynthesis Pathway	Reactome	10.3180/R-HSA-191273.7	Submap and main map
	WikiPathways	WP4718	
	Literature	Annotated on the map	
Fatty Acid and Cholesterol Transporters	WikiPathways	WP5061	Submap and main map
	WikiPathways	WP5304	
	KEGG	map04979	
	Literature	Annotated on the map	
Glucose metabolism	Atlas of Inflamation Resolution	AIR	Submap and main map
	WikiPathways	WP534	
	WikiPathways	WP134	
	WikiPathways	WP500	
	Literature	Annotated on the map	
Glucagon signaling	Literature	Annotated on the map	Submap and main map
Insulin signaling	Ageing Map	Ageing Map	Submap and main map
	Literature	Annotated on the map	
Fatty acid omega-oxidation	Wikipathways	WP206	Main map
	Literature	Annotated on the map	
Peroxisomal beta-oxidation	Wikipathways	WP1941	Main map
	Literature	Annotated on the map	
Triacylglyceride Synthesis	Wikipathways	WP325	Main map
	Literature	Annotated on the map	
Mitochondrial Metabolism Pathways	Parkinson's Disease Map	PDMap	Submap
	COVID19 Disease Map	COVID-19 Disease Map	
	WikiPathways	WP5175	
	WikiPathways	WP357	
	Literature	Annotated on the map	
Gene regulatory network	Wikipathways	WP2882	Main map
	Literature	Annotated on the map	
Lipid droplet	WikiPathways	WP3901	Submap
