# Supplementary material for: Unlocking liver physiology: comprehensive pathway maps for mechanistic understanding
Source: Front Toxicol. 2025 Jul 7;7:1619651. doi: 10.3389/ftox.2025.1619651 (PMC12277266; doi:10.3389/ftox.2025.1619651)
Supplement: Supplementary file 1 [file Supplementaryfile1.zip › Supplementary Information/Supplementary text.docx]

Unlocking liver physiology: comprehensive pathway maps for mechanistic understanding

# Supplementary Information

# Supplementary Methods

## Physiological maps cross-comparison analysis

The comparative analysis of the liver physiological maps (PMs) involved data extraction, statistical summary of entity frequency, and functional enrichment to characterize the molecular composition and functional relationships between the Liver Lipid Metabolism (LiverLipidPM) and Liver Bile Secretion PMs (LiverBilePM).

Molecular components from both PMs were retrieved using the minervar R package (Gawron et al., 2023) to interface with the MINERVA platform API (<https://ontox.elixir-luxembourg.org/minerva/api/>) (Hoksza et al., 2019). The data were extracted from the main map and submaps (identifiers 552-559 for the LiverLipidPM and 560-569 for the LiverBilePM). For each map, both molecular elements and reaction data were retrieved and processed to retain essential information including component names and types. Duplicate entries were removed to ensure accurate quantification of unique molecular entities.

Gene lists were compiled by filtering molecular elements to include only those classified as "Gene", "Protein", or "RNA" types, for the reason that these node types were named after their related HGNC approved symbols. These curated gene lists served as the foundation for subsequent comparative and functional analyses. Descriptive statistics were generated to characterize the molecular composition of each PM. Element frequency distributions were calculated and visualized on Supplementary Figure 1 A and B.

Comparative analysis between the two PMs was performed using set operations to identify shared and unique molecular components. Visualization of the overlap and unique gene sets between the LiverLipidPM and LiverBilePM was done using a Venn diagram (Supplementary Figure 1 C). The intersection and set differences were calculated to quantify shared genes, genes unique to the LiverLipidPM and genes unique to the LiverBilePM. These gene lists can be found in the Supplementary Information > *analysis_scripts_and_outputs* > *output* > *tables_and_lists* folder.

To elucidate the biological significance of the comparative results, pathway enrichment analysis was performed using the ReactomePA package (Yu & He, 2016). Gene symbols were converted to Entrez IDs using the org.Hs.eg.db (Carlson, 2017) annotation package, with unmapped genes excluded from the analysis. Separate enrichment analyses were conducted for three gene sets: shared genes between both maps, genes unique to the LiverLipidPM, and genes unique to the LiverBilePM. These gene lists can be found in the Supplementary Information > *analysis_scripts_and_outputs* > *output* > *tables_and_lists* folder.

Reactome pathway enrichment was performed using the enrichPathway function with a p-value cutoffs of 0.05 and q-value cutoffs of 0.1 for shared gene sets, and p-value cutoffs of 0.05 for unique gene sets.. Results were converted back to human-readable gene symbols (HGNC approved symbols) for interpretation. Enrichment results from all three gene sets can be found at Supplementary Information > *analysis_scripts_and_outputs* > *output* > *tables_and_lists* folder, and the top 5 enriched pathways for each gene set were visualized in a bubble plot (Supplementary Figure 2 D).

A reproducible script for this analysis can be found at Supplementary Information > *analysis_scripts_and_outputs* > *scripts > 1_map_analysis.R.*

## AOP Networks and physiological map’s cross-comparison analysis

Gene lists from established adverse outcome pathway (AOP) networks were obtained their respective authors for comparative analysis with the liver PMs. The steatosis AOP network gene list was derived from Verhoeven et al. (2024), while the cholestasis AOP network gene list was obtained from Van Ertvelde et al. (2023). The raw data can be found in the respective original publication’s supplementary materials, while the gene lists used in this work can be found in Supplementary Information > *analysis_scripts_and_outputs* > *data* folder. These AOP networks represent well-characterized toxicological pathways relevant to liver pathophysiology and provided a framework for evaluating the coverage and specificity of the physiological maps.

Two independent comparative analyses were performed: the LiverLipidPM was compared against the steatosis AOP network, and the LiverBilePM was compared against the cholestasis AOP network. For each comparison, set operations were applied to identify shared genes, genes unique to the PM, and genes unique to the AOP network. Venn diagrams were generated to visualize the overlap patterns and quantify the degree of molecular concordance between physiological and toxicological representations (Supplementary Figure 2 A and B).

Pathway enrichment analysis was conducted for each of the three gene sets derived from both comparisons (shared, PM-unique, and AOP-unique genes). Gene symbols were converted to Entrez IDs using org.Hs.eg.db, with unmapped genes excluded. Reactome pathway enrichment was performed using ReactomePA with p-value cutoffs of 0.05 and q-value cutoffs of 0.1 for shared gene sets, and p-value cutoffs of 0.05 for unique gene sets. The top 5 enriched terms for each group in each analysis were visualized in a bubble plot (Supplementary Figure 2 C and D), and the complete enrichment results can be found at Supplementary Information > *analysis_scripts_and_outputs* > *output* > *tables_and_lists* folder.

Enriched genes unique to each physiological map were extracted from the enrichment results for subsequent analysis. Gene identifiers were parsed from the enrichment output, deduplicated, and saved as individual gene lists to support downstream compound-target identification.

A reproducible script for this analysis can be found at Supplementary Information > *analysis_scripts_and_outputs* > *scripts > 2_aop_analysis.R.*

## Compound-target interaction querying

To identify potential molecular initiating events (MIE) targets within the liver PMs, which might be missed in the respective AOP network, a comprehensive chemical compound-target interaction query was performed using the MINERVA platform's integrated functions to explore DrugBank [(Knox et al. 2024)](https://www.zotero.org/google-docs/?KPVRgl) and ChEMBL [(Zdrazil et al. 2024)](https://www.zotero.org/google-docs/?EhmbVx) databases. Map bioentities were retrieved from both the LiverLipidPM and LiverBilePM in the same way as in section *1.1 Physiological maps cross-comparison analysis*.

Reference gene lists containing unique genes for each PM were loaded from previously generated enrichment analysis results. Map bioentities were filtered to include only those present in the respective reference gene lists, focusing the analysis on genes unique to each physiological process. chemical-target searches were conducted through programmatic queries to the MINERVA API using the filtered bioentity identifiers as search targets. The resulting datasets were filtered to retain only interactions involving genes present in the unique reference lists for each PM and can be found at Supplementary Information > *analysis_scripts_and_outputs* > *output* > *tables_and_lists* folder.

A reproducible script for this analysis can be found at Supplementary Information > *analysis_scripts_and_outputs* > *scripts > 3_chemicals_search.R.*

## Data overlay preparation for visualization in MINERVA

To assess the coverage of AOP networks within the PMs, gene lists from both analyzed AOP networks were processed and prepared for visualization as overlays on the MINERVA platform. For that, we provide a comprehensive and reproducible R script for processing the gene lists from AOP networks for visualization, which can be found at Supplementary Information > *analysis_scripts_and_outputs* > *scripts > 4_overlays.R*. The processed data were formatted according to MINERVA overlay specifications, including standardized headers containing version information, descriptive names, detailed descriptions, and bibliographic references. The resulting overlay files were saved as tab-separated text files compatible with the MINERVA platform's visualization engine.

It is important to mention that different data types will have different treatment requirements for better visualization in MINERVA, and this should be done in a fit-for-purpose fashion. We recommend the reader to explore the MINERVA platform user’s manual at <https://minerva.pages.uni.lu/doc/> for detailed information. We also provide a reproducible R script for processing quantitative transcriptomics data as an example in the second half of the script found at Supplementary Information > *analysis_scripts_and_outputs* > *scripts > 4_overlays.R*.

## Software

All the analysis were done using R version 4.4.0 (2024-04-24 ucrt) (R Core Team, 2024) and R Studio version 2023.12.1.402 (Ocean Storm) (Posit team, 2024).

# Supplementary Figures


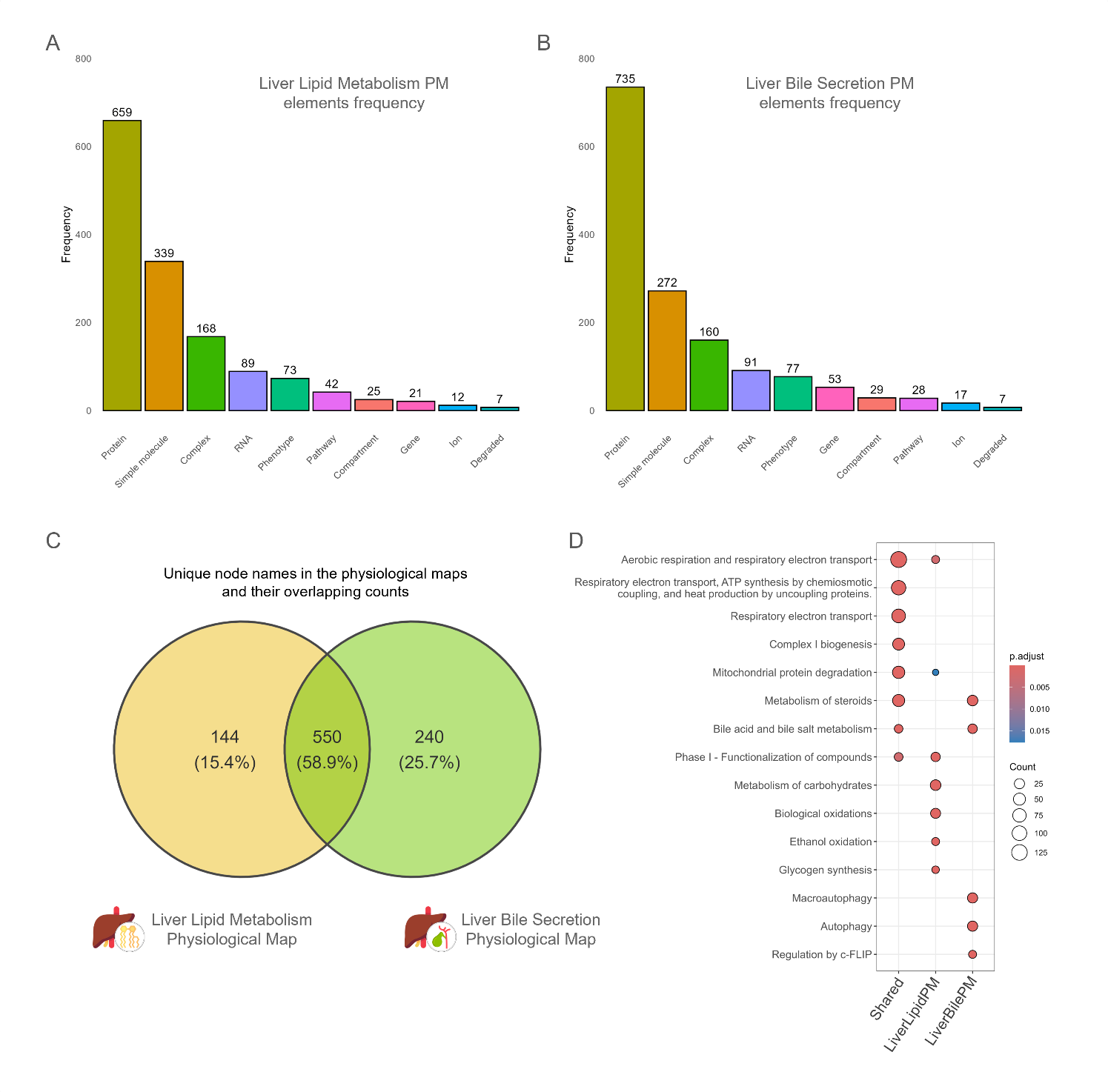


***Supplementary Figure 1.*** *Liver Lipid Metabolism Physiological Map (LiverLipidPM) and Liver Bile Secretion Physiological Map (LiverBilePM) cross-comparison. A – LiverLipidPM entity frequency. B – LiverBilePM entity frequency. C – Unique and overlapping entity names in the physiological maps and their counts. D – Reactome pathways enrichment of shared and unique entity names for the LiverLipidPM and LiverBilePM.*


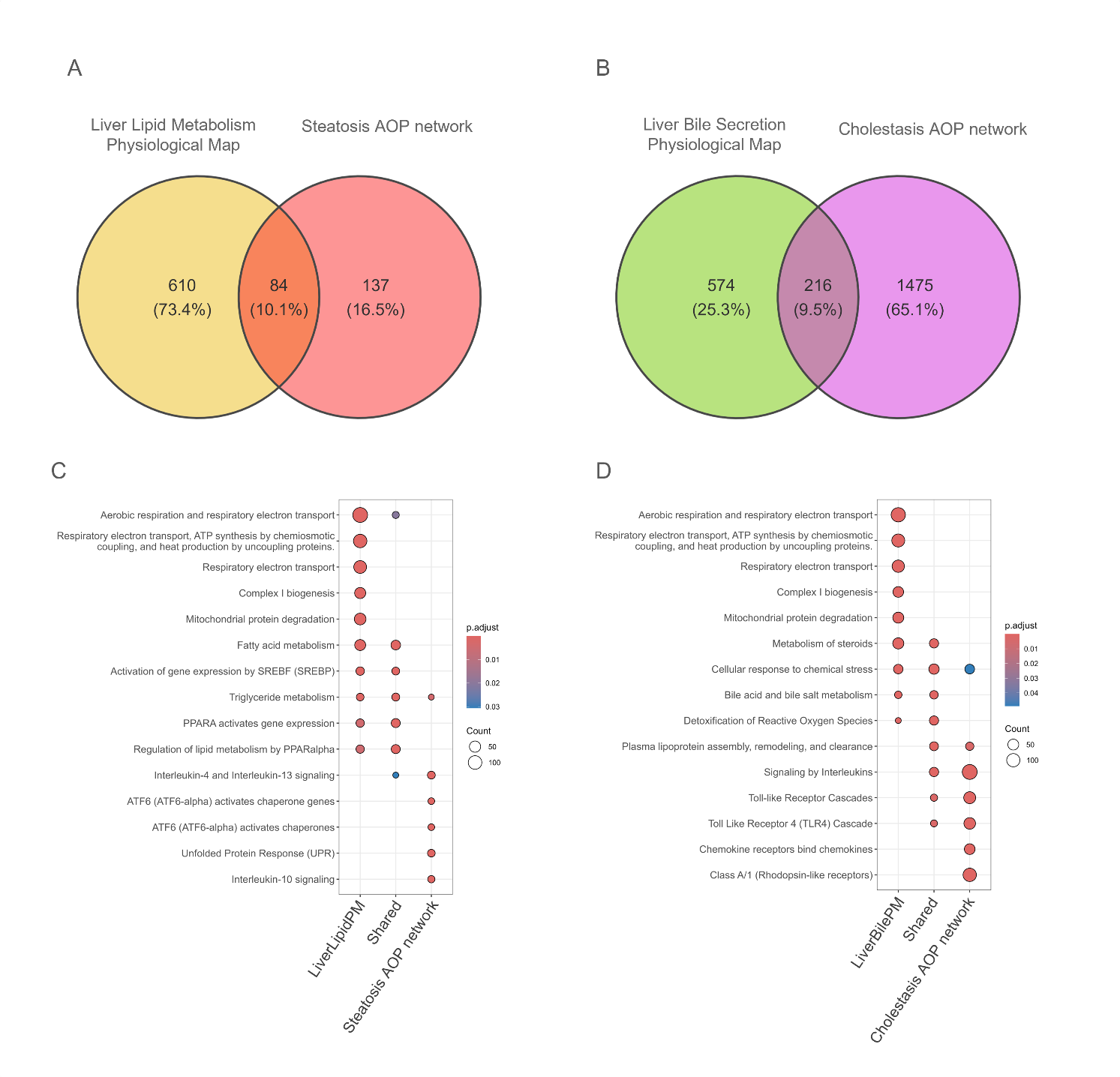


***Supplementary Figure 2.*** *Adverse Outcome Pathway (AOP) networks cross-comparison against their relative Physiological Maps (PM). A – Unique and overlapping entity names in the LiverLipidPM and Steatosis AOP network and their counts. B – Unique and overlapping entity names in the LiverBilePM and Cholestasis AOP network and their counts. C – Reactome pathways enrichment of shared and unique entity names for the LiverLipidPM and Steatosis AOP network. D – Reactome pathways enrichment of shared and unique entity names for the LiverBilePM and Cholestasis AOP network.*

# Supplementary References

Carlson, M. 2017. "Org.Hs.eg.db" [Computer software]. Bioconductor. https://doi.org/10.18129/B9.BIOC.ORG.HS.EG.DB

Gawron, P., Smula, E., Schneider, R., & Ostaszewski, M. 2023. "Exploration and comparison of molecular mechanisms across diseases using MINERVA Net." Protein Science, 32(2), e4565. https://doi.org/10.1002/pro.4565

Hoksza, D., Gawron, P., Ostaszewski, M., Smula, E., & Schneider, R. 2019. "MINERVA API and plugins: Opening molecular network analysis and visualization to the community." Bioinformatics, 35(21), 4496–4498. https://doi.org/10.1093/bioinformatics/btz286

Posit team. 2024. "RStudio: Integrated Development Environment for R." [R]. Posit Software. https://posit.co/download/rstudio-desktop/

R Core Team. 2024. "R: A Language and Environment for Statistical Computing." [R]. https://www.R-project.org/

Van Ertvelde, J., Verhoeven, A., Maerten, A., Cooreman, A., Santos Rodrigues, B. D., Sanz-Serrano, J., Mihajlovic, M., Tripodi, I., Teunis, M., Jover, R., Luechtefeld, T., Vanhaecke, T., Jiang, J., & Vinken, M. 2023. "Optimization of an adverse outcome pathway network on chemical-induced cholestasis using an artificial intelligence-assisted data collection and confidence level quantification approach." Journal of Biomedical Informatics, 145, 104465. https://doi.org/10.1016/j.jbi.2023.104465

Verhoeven, A., Van Ertvelde, J., Boeckmans, J., Gatzios, A., Jover, R., Lindeman, B., Lopez-Soop, G., Rodrigues, R. M., Rapisarda, A., Sanz-Serrano, J., Stinckens, M., Sepehri, S., Teunis, M., Vinken, M., Jiang, J., & Vanhaecke, T. 2024. "A quantitative weight-of-evidence method for confidence assessment of adverse outcome pathway networks: A case study on chemical-induced liver steatosis." Toxicology, 505, 153814. https://doi.org/10.1016/j.tox.2024.153814

Yu, G., & He, Q.-Y. 2016. "ReactomePA: An R/Bioconductor package for reactome pathway analysis and visualization". Molecular BioSystems, 12(2), 477–479. https://doi.org/10.1039/C5MB00663E
