# Supplementary figures and images for: Unlocking liver physiology: comprehensive pathway maps for mechanistic understanding
Source: Front Toxicol. 2025 Jul 7;7:1619651. doi: 10.3389/ftox.2025.1619651 (PMC12277266; doi:10.3389/ftox.2025.1619651)

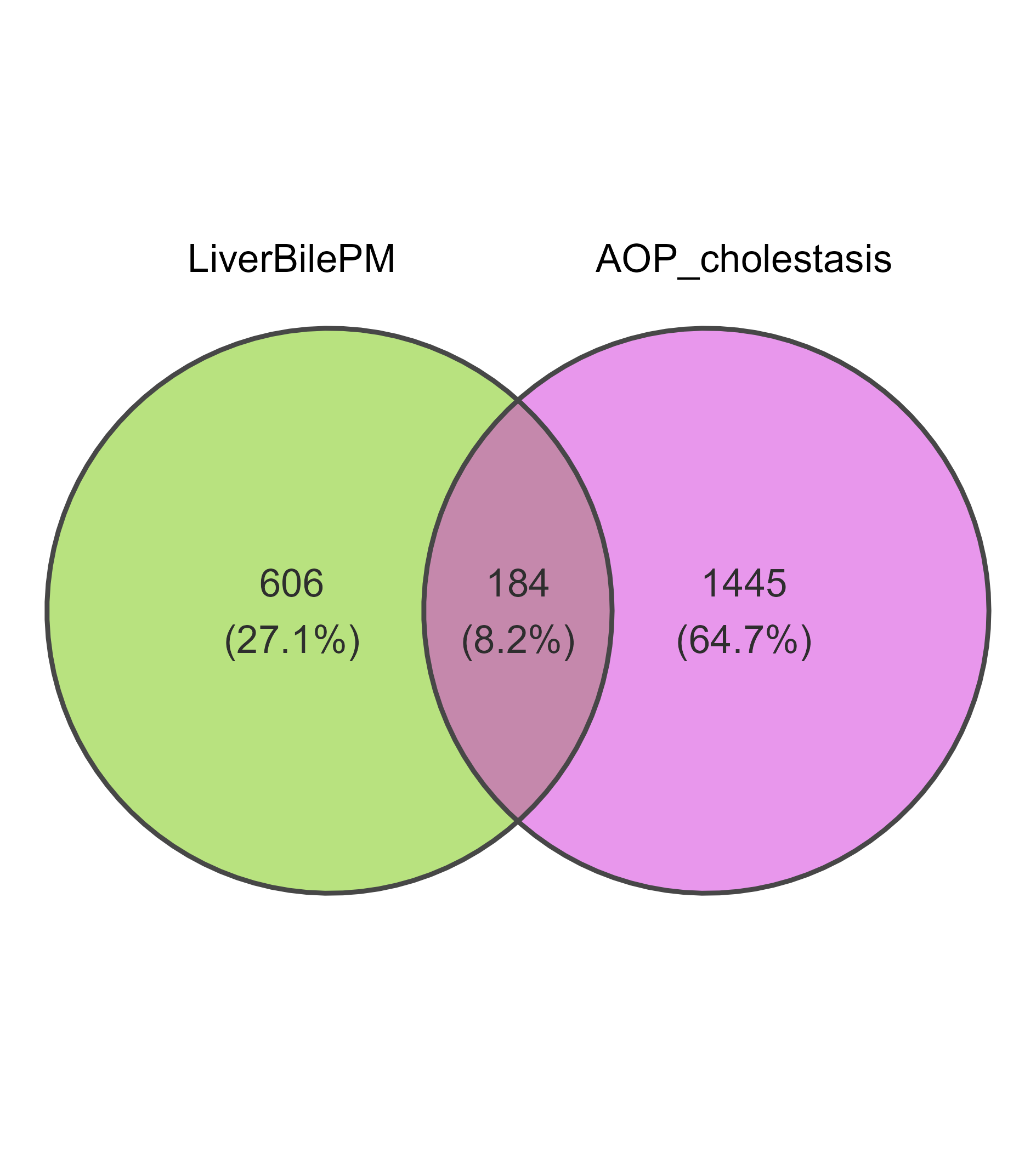

Supplement: Supplementary file 1 [file Supplementaryfile1.zip › Supplementary Information/analysis_scripts_and_outputs/output/figures/cholestasis_aop_overlap_with_liverbilepm.png]

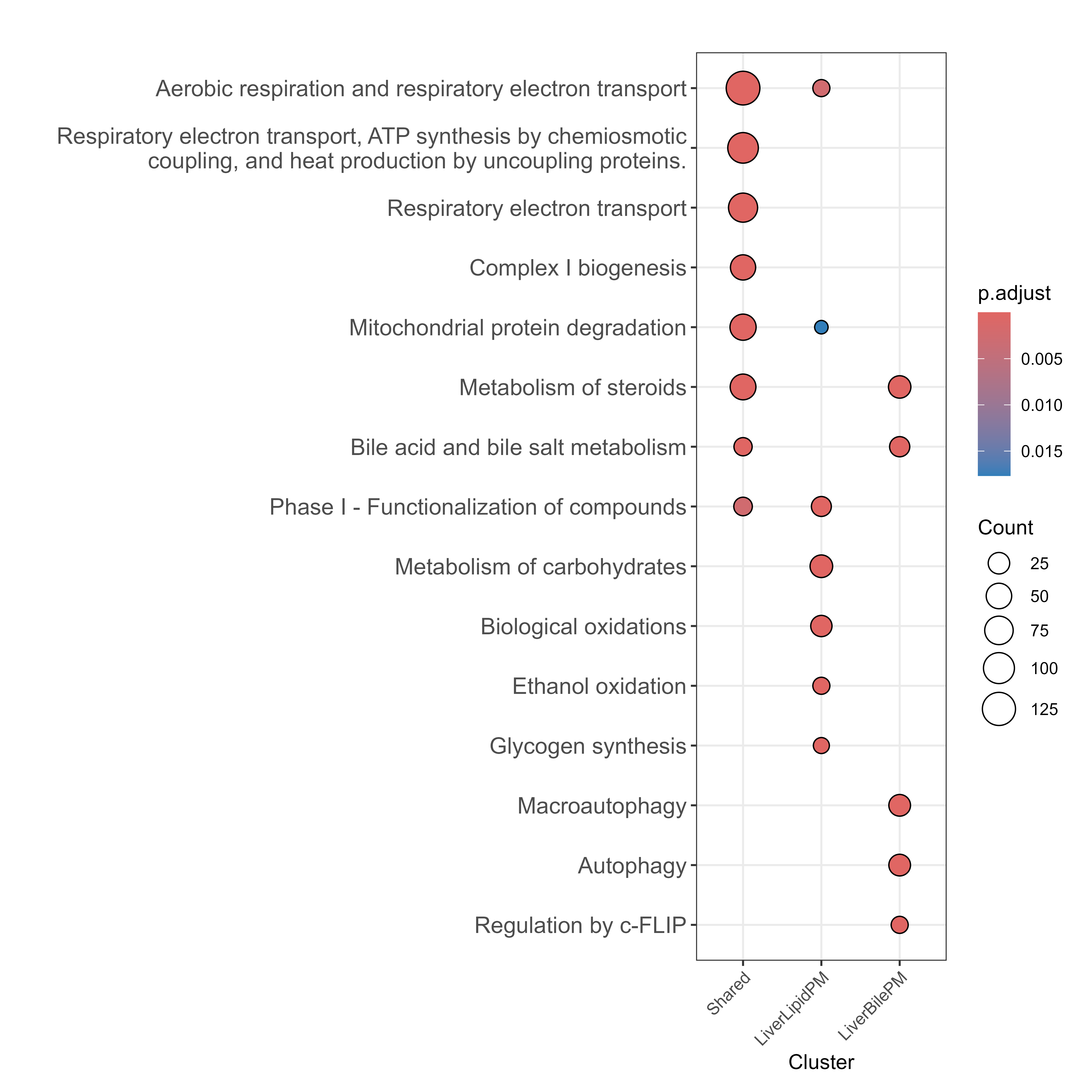

Supplement: Supplementary file 1 [file Supplementaryfile1.zip › Supplementary Information/analysis_scripts_and_outputs/output/figures/crossmap_enrichment_comparison_plot.png]

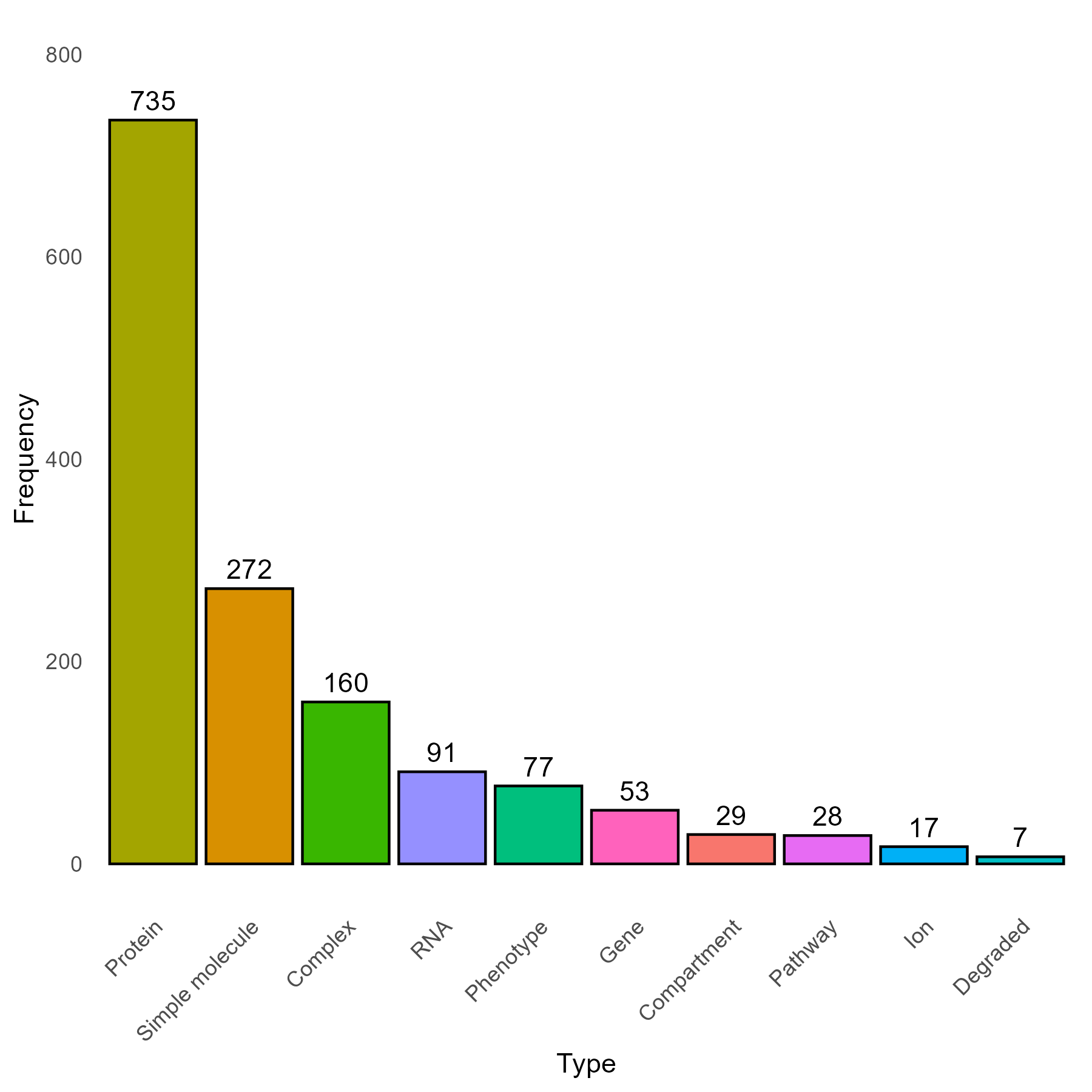

Supplement: Supplementary file 1 [file Supplementaryfile1.zip › Supplementary Information/analysis_scripts_and_outputs/output/figures/element_frequency_liverbilepm_barplot.png]

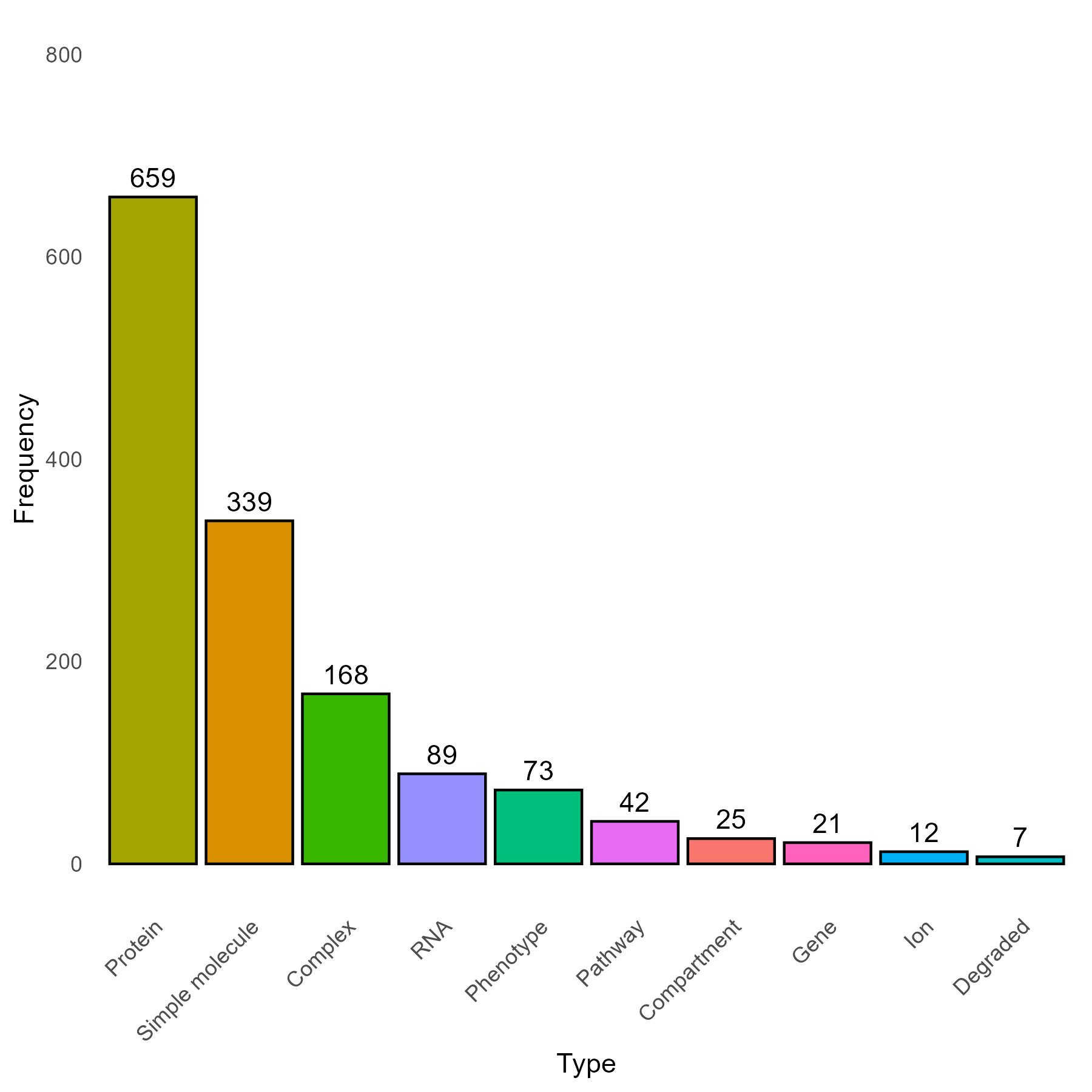

Supplement: Supplementary file 1 [file Supplementaryfile1.zip › Supplementary Information/analysis_scripts_and_outputs/output/figures/element_frequency_liverlipidpm_barplot.png]

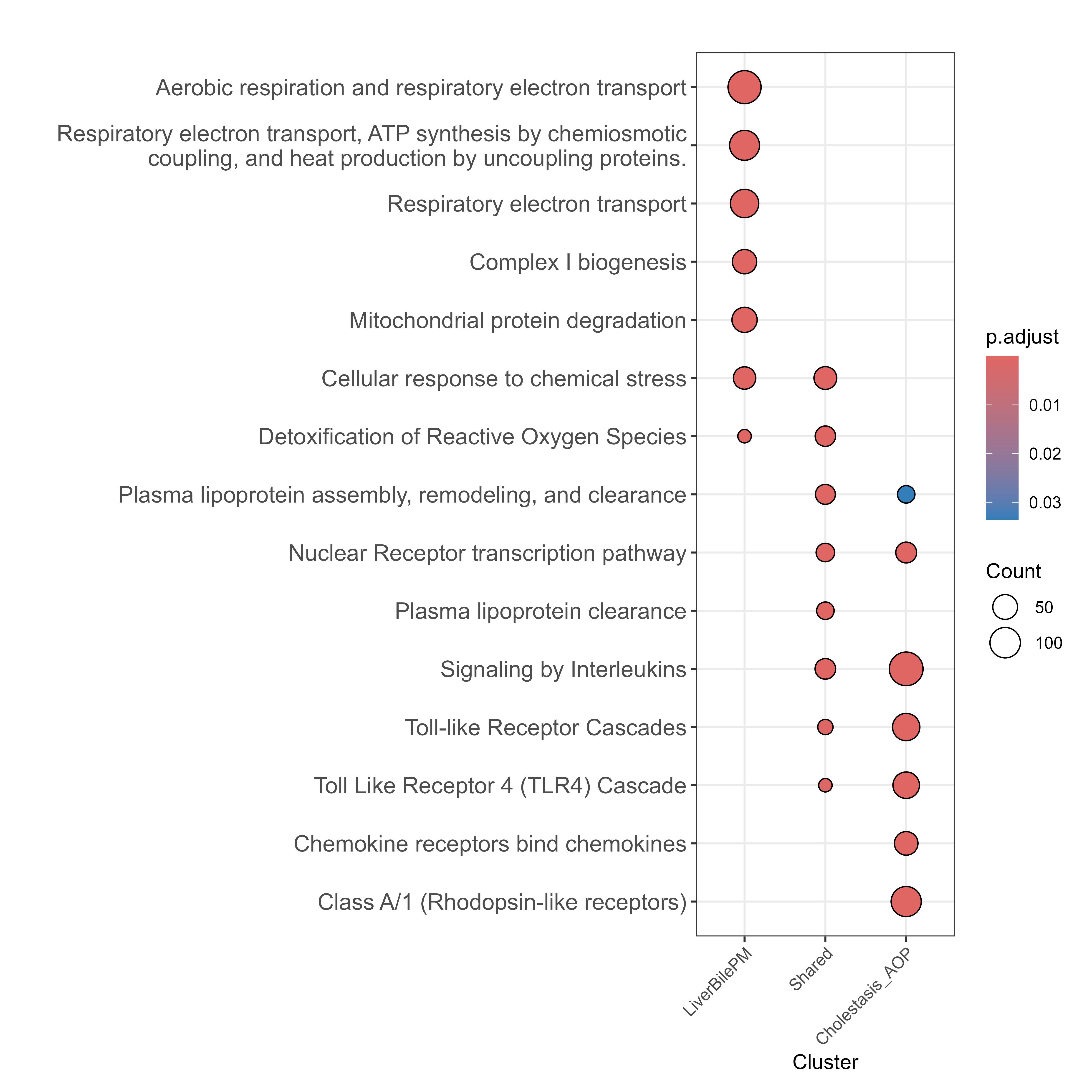

Supplement: Supplementary file 1 [file Supplementaryfile1.zip › Supplementary Information/analysis_scripts_and_outputs/output/figures/enrichment_cholestasis_aop_comparison_with_liverbilepm.png]

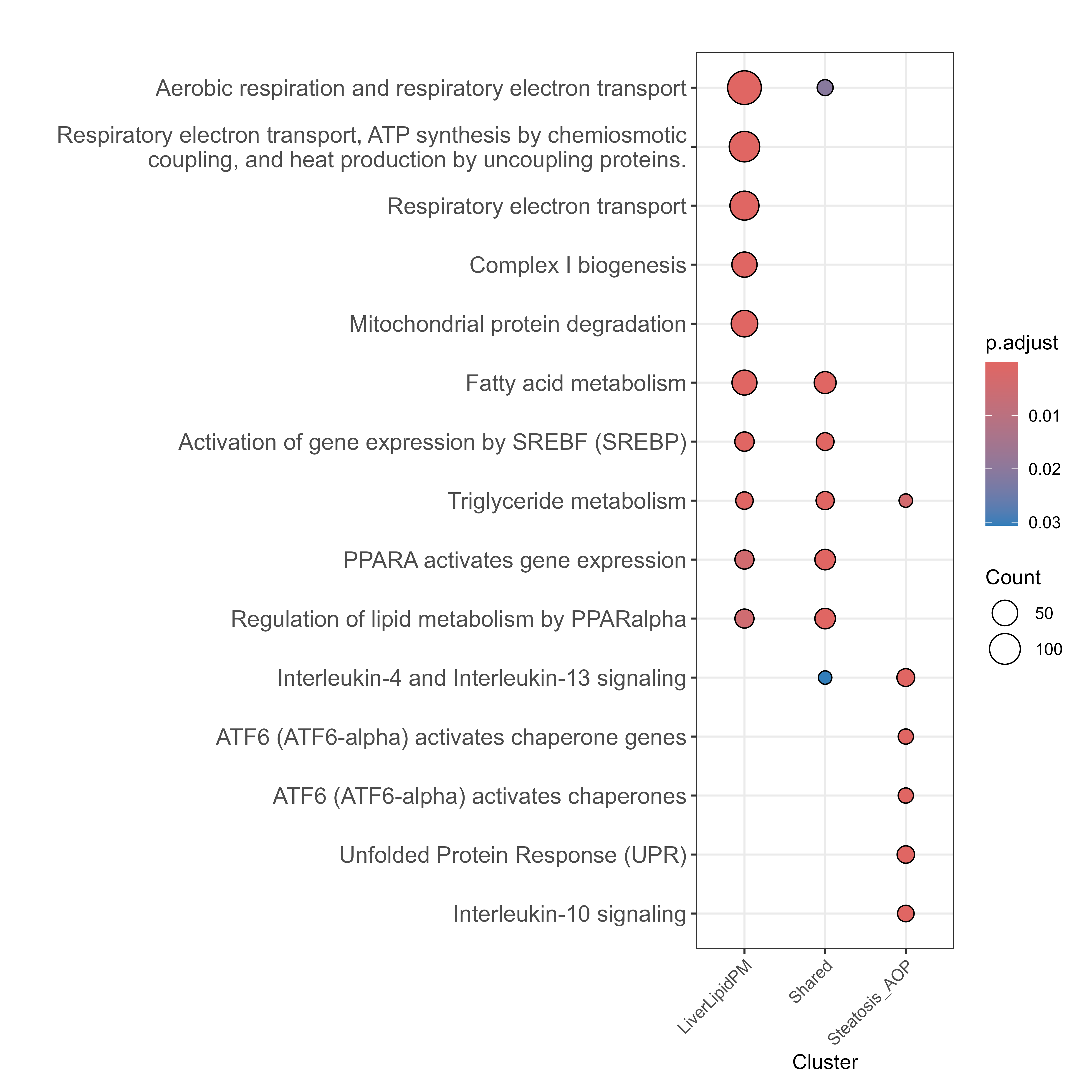

Supplement: Supplementary file 1 [file Supplementaryfile1.zip › Supplementary Information/analysis_scripts_and_outputs/output/figures/enrichment_steatosis_aop_comparison_with_liverlipidpm.png]

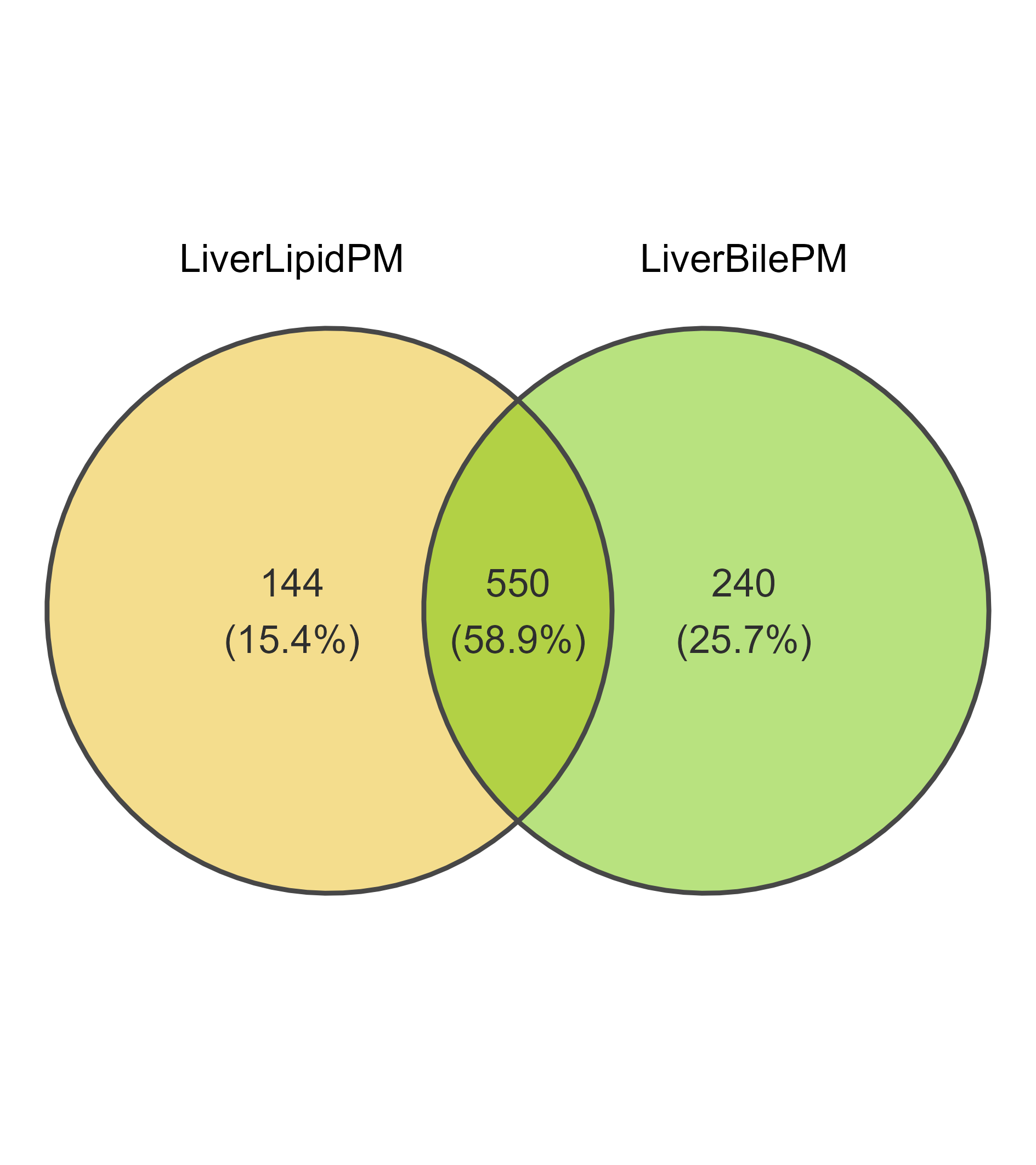

Supplement: Supplementary file 1 [file Supplementaryfile1.zip › Supplementary Information/analysis_scripts_and_outputs/output/figures/maps_entity_overlap.png]

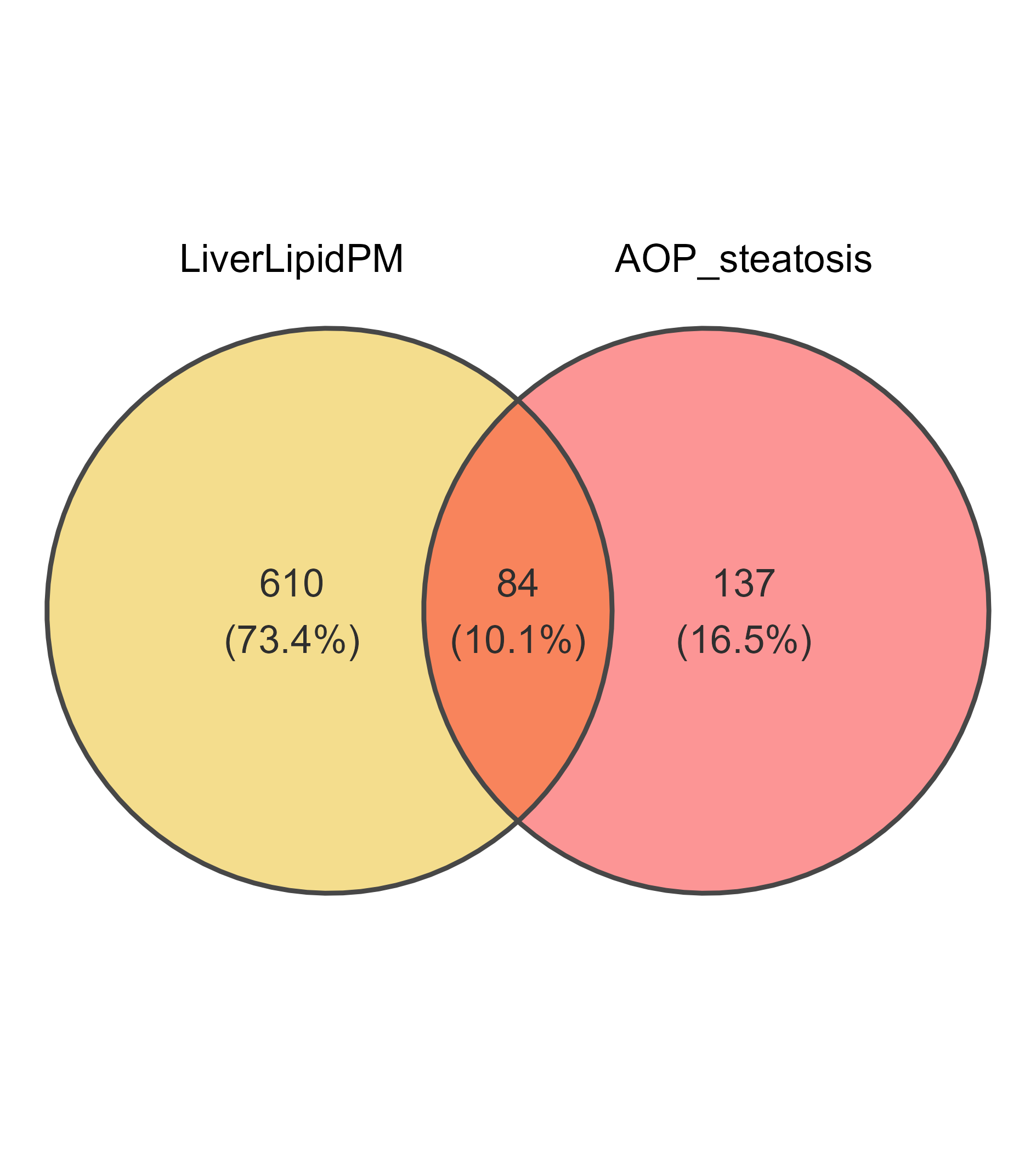

Supplement: Supplementary file 1 [file Supplementaryfile1.zip › Supplementary Information/analysis_scripts_and_outputs/output/figures/steatosis_aop_overlap_with_liverlipidpm.png]

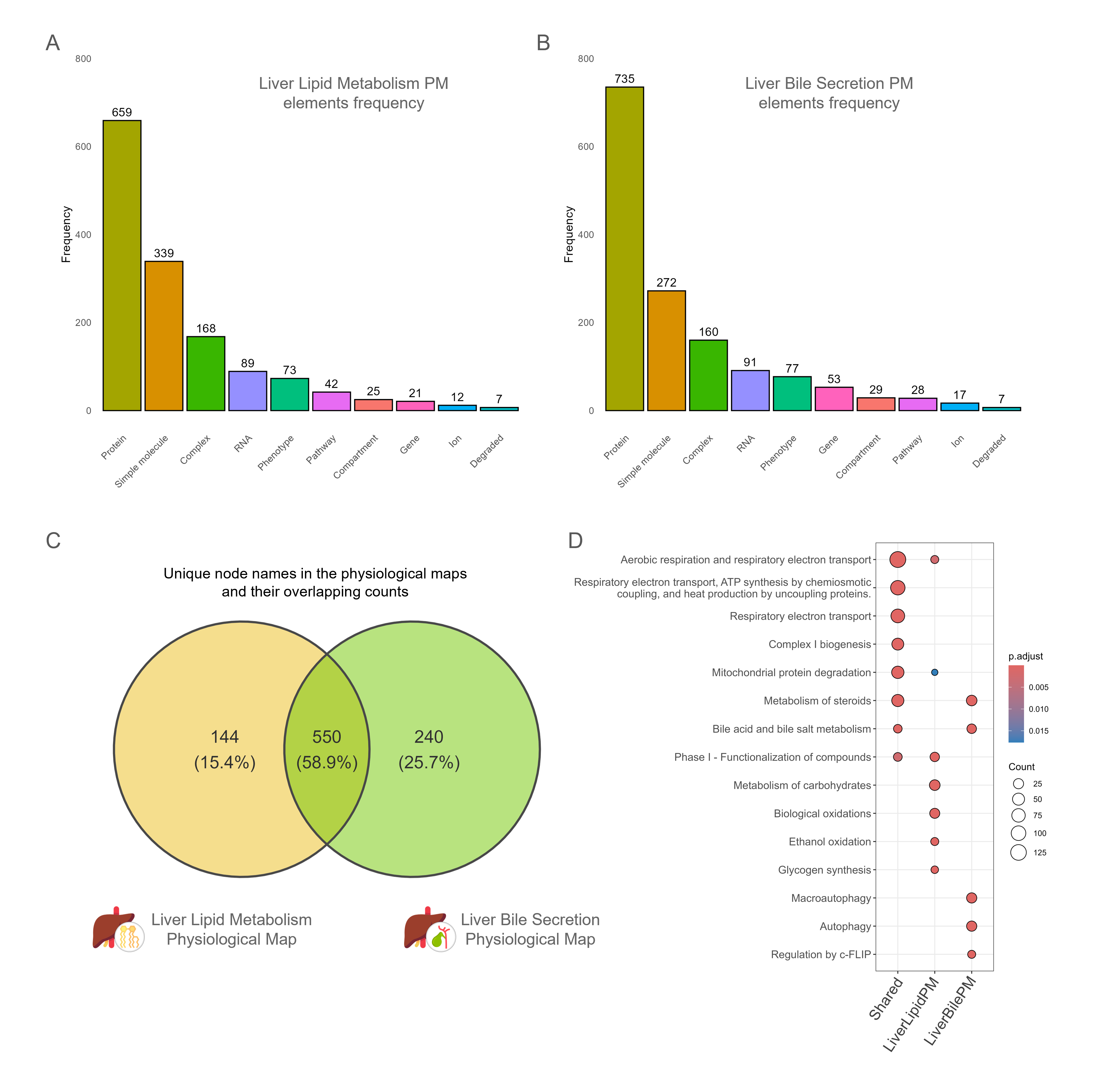

Supplement: Supplementary file 1 [file Supplementaryfile1.zip › Supplementary Information/Supplementary Figure S1.png]

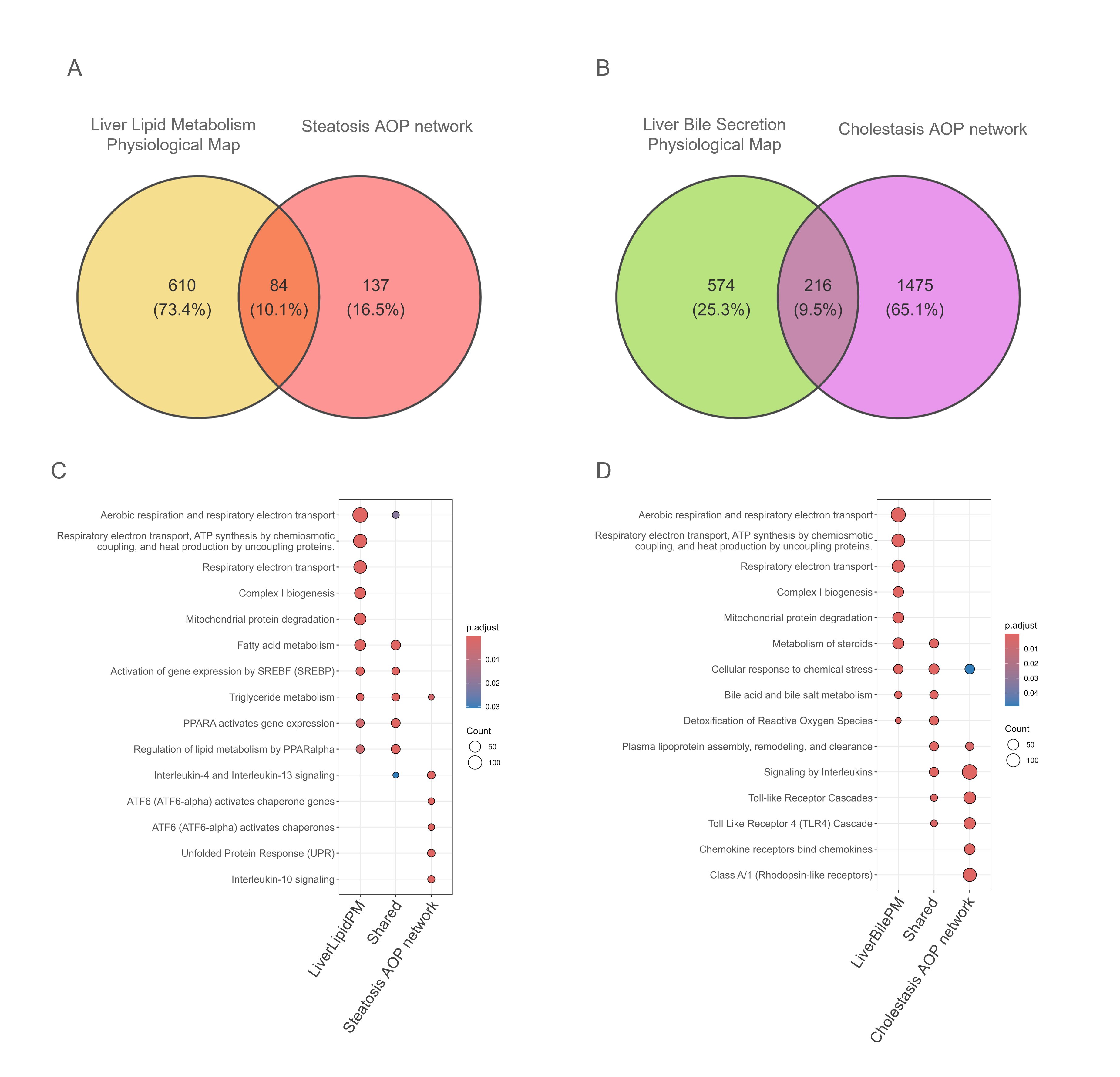

Supplement: Supplementary file 1 [file Supplementaryfile1.zip › Supplementary Information/Supplementary Figure S2.png]
